# PPAR-γ/NF-kB/AQP3 axis in M2 macrophage orchestrates lung adenocarcinoma progression by upregulating IL-6

**DOI:** 10.1038/s41419-024-06919-9

**Published:** 2024-07-26

**Authors:** Guofu Lin, Lanlan Lin, Xiaohui Chen, Luyang Chen, Jiansheng Yang, Yanling Chen, Danwen Qian, Yiming Zeng, Yuan Xu

**Affiliations:** 1 Fujian Provincial Clinical Research Center of Interventional Respirology, The Second Affiliated Hospital of Fujian Medical University, Quanzhou, Fujian Province 362000 China; 2 Department of Pulmonary and Critical Care Medicine, The Second Affiliated Hospital of Fujian Medical University, Quanzhou, Fujian Province 362000 China; 3 Fujian Provincial Key Laboratory of Lung Stem Cells, Ouanzhou, Fujian Province 362000 China; 4 Department of Thoracic Surgery, The Second Affiliated Hospital of Fujian Medical University, Quanzhou, Fujian province 362000 China; 5 Clinical Research Center, The Second Affiliated Hospital of Fujian Medical University, Quanzhou, Fujian Province 362000 China; 6 The Tumor Immunogenomics and Immunosurveillance (TIGI) Lab, UCL Cancer Institute, London, UK

**Keywords:** Lung cancer, Tumour immunology

## Abstract

Aquaporin 3 (AQP3), which is mostly expressed in pulmonary epithelial cells, was linked to lung adenocarcinoma (LUAD). However, the underlying functions and mechanisms of AQP3 in the tumor microenvironment (TME) of LUAD have not been elucidated. Single-cell RNA sequencing (scRNA-seq) was used to study the composition, lineage, and functional states of TME-infiltrating immune cells and discover AQP3-expressing subpopulations in five LUAD patients. Then the identifications of its function on TME were examined in vitro and in vivo. AQP3 was associated with TNM stages and lymph node metastasis of LUAD patients. We classified inter- and intra-tumor diversity of LUAD into twelve subpopulations using scRNA-seq analyses. The analysis showed AQP3 was mainly enriched in subpopulations of M2 macrophages. Importantly, mechanistic investigations indicated that AQP3 promoted M2 macrophage polarization by the PPAR-γ/NF-κB axis, which affected tumor growth and migration via modulating IL-6 production. Mixed subcutaneous transplanted tumor mice and Aqp3 knockout mice models were further utilized, and revealed that AQP3 played a critical role in mediating M2 macrophage polarization, modulating glucose metabolism in tumors, and regulating both upstream and downstream pathways. Overall, our study demonstrated that AQP3 could regulate the proliferation, migration, and glycometabolism of tumor cells by modulating M2 macrophages polarization through the PPAR-γ/NF-κB axis and IL-6/IL-6R signaling pathway, providing new insight into the early detection and potential therapeutic target of LUAD.

## Background

Lung cancer is a common and lethal tumor that accounts for the largest cancer-related mortality worldwide [1]. In the United States, lung cancer incidence and mortality were recorded as 2.2 million and 1.8 million cases, accounting for 11.4% of all cancer cases and 18.1% of all cancer deaths, respectively [2]. In 2016, there were an estimated 828,000 new cases and 657,000 deaths from lung cancer in China [3]. Lung adenocarcinoma (LUAD), the most frequent non-small cell lung cancer (NSCLC) subtype, accounts for 40% of lung cancer incidences [4]. Despite surgery being the standard treatment, the recurrence rates remain high, leading to poor 5-year survival rates [5, 6]. In recent years, targeted therapy and immunotherapy have shown promise for LUAD patients; however, drug resistance and recurrence progression of tumor remains the major challenge. Thus, understanding tumor development is vital to improving lung cancer patients’ outcomes.

The tumor microenvironment (TME) consists of various cells that interact with tumor cells and regulate biological processes [7]. Macrophages, one of the most important subpopulation in TME, can respond to signals in the microenvironment by changing their functional phenotypes [8]. M1 macrophages are activated through the classical activation pathway and play a role in recruiting cytotoxic T cells and natural killer cells, acting directly on tumor cells [9]. M2 macrophages enhance tissue remodeling and wound healing by the alternative activation pathway [10, 11]. Tumor-associated macrophages (TAMs), mainly characterized as M2-type macrophages, could promote disease progression and deteriorate patients’ prognosis [12]. Suppressing M2 polarization and promoting M2 to M1 repolarization are two TAM-targeted anti-tumor adjuvant treatment methods [13, 14]. Therefore, a comprehensive study of the mechanism of M2 macrophages on LUAD is of clinical significance for targeting TAMs during anti-tumor adjuvant therapy [15]. Several studies have identified different TAMs subpopulations using single-cell RNA sequencing and found that some TAMs subpopulations expressed M1 and M2 macrophages surface marker genes, indicating the existence of an intermediate TAMs subpopulation between M1 and M2 macrophages [16, 17]. However, the underlying regulatory mechanism and its relationship with M2 macrophage polarization in LUAD remains incompletely clarified.

Aquaporin-3 (AQP3), a member of the aquaporin family, is expressed in normal lung tissue’s type II alveolar epithelium and trachea and bronchi epithelial cells [18]. Previous studies have demonstrated that knocking down AQP3 in lung adenocarcinoma cells could not only inhibit tumor proliferation and migration [19, 20], but also impact biological processes like epithelial-mesenchymal transition and neovascularization, thereby playing an important regulatory role in tumor progression [21]. Our previous studies have shown that AQP3 was involved in the tumorigenesis and progression of LUAD [22]. However, the expression levels, biological functions and potential mechanisms in the microenvironment of LUAD are still unknown. In the present study, we have systematically investigated the clinical relevance and anti-cancer efficacy of AQP3 in LUAD microenvironment in vitro and in vivo.

## Methods

### Patient specimens

Human LUAD cancer and adjacent non-tumor tissue samples for RT-qPCR analysis were obtained from a total of 112 patients who underwent surgical resection in the Department of Thoracic Surgery, The Second Affiliated Hospital of Fujian Medical University. Additionally, 100 paraffin-embedded LUAD specimens and adjacent non-tumor specimens from 2013 to 2018 were included in this study. All patients underwent surgery without receiving any anti-tumor treatment before the operation. The study was approved by the institutional ethics committee (approval No. 2022-88) and was performed according to the principles of the Declaration of Helsinki. All participants provided informed written consent.

### Cell lines and culture conditions

The human lung carcinoma cell line (A549), mouse Lewis lung cancer cell line (LLC), human monocyte cell line (THP-1), and leukemia cells in mouse macrophage (RAW264.7) were acquired from ATCC (Manassas, USA). The cells were grown in RPMI-1640 medium supplemented with 10% fetal bovine serum (Invitrogen, USA) and 1% penicillin-streptomycin (Gibco, USA) at 37 °C in a 5% CO_2_ atmosphere.

### Establishment of the M1- and M2-polarized macrophages model and TAMs model

THP-1 cells were stimulated with PMA (100 ng/mL) for 24 h to differentiate monocytes into macrophages [23, 24]. To establish M1 and M2 polarization of macrophages in PMA-treated THP-1 cells and RAW264.7, respectively, IFN-γ (20 ng/mL) + LPS (100 ng/mL) and IL-4 (20 ng/mL) + IL-13 (20 ng/mL) were used for stimulation for 48 h [25].

We used the 24 transwell co-culture system (BD Biosciences, USA) with a pore size of 0.4 μm to establish a TAMs model for the non-contact co-culture. Specifically, 2 × 10^4^/mL lung cancer cells (A549 or LLC) were seeded in the upper chamber, while 5 × 10^5^/mL M0 macrophages (PMA-treated THP-1 macrophages or RAW264.7) were seeded in the lower chamber. This setup replicated tumor-macrophage interactions in vitro [26].

### Quantitative RT-PCR (RT-qPCR)

RT-qPCR was performed according to standard protocols. To extract total RNA from LUAD specimens and cultured cells, TRIzol® Reagent (Invitrogen, USA) was used. The extracted RNA was then transcribed into cDNA using the PrimeScript^TM^ RT reagent Kit (Takara, Japan). Real-time quantitative PCR was performed using the TB Green Premix Ex Taq II (Takara, Japan) with the 7500 Real-Time PCR machine (Applied Biosystems). The relative gene expression was estimated using the 2^-ΔΔCt^ technique after being adjusted to the expression of a housekeeping gene (GAPDH). The sequences of specific primers used in the experiment are presented in Table S1.

### Western blot

Western blot was performed as previously described [27]. Briefly, tissues and cells were lysed in RIPA buffer (Beyotime, China), and protein concentration was quantified using a BCA protein assay kit (PC0020, Solarbio, China). The samples were loaded onto 8 or 10% gels and transferred to PVDF membranes (Millipore, USA). The membranes were blocked with a 5% milk solution in TBST for 2 h at room temperature before incubating them with primary antibodies at 4 °C overnight. The primary antibodies used were Anti-AQP3 (0.5 ug/mL, ab125219, Abcam), Anti-CD163 (1:1000, ab182422, Abcam), Anti-IL-6 (1:1000, GTX110527, GeneTex), Anti-IL-6R (1:1000, DF6466, Affinity), Anti-STAT3 (1:1000, ab68153, Abcam), Anti-p-STAT3 (1:1000, ab76315, Abcam), Anti-GAPDH (1:1000, ab8245, Abcam), and Anti-β-actin (1:3000, ab8227, Abcam). The membranes were treated at room temperature for 1 h with an HRP-conjugated secondary antibody. The Western blot was visualized using enhanced chemiluminescence, and image acquisition was detected using ImageQuant LAS 4000 (GE Healthcare, UK).

### Hematoxylin and eosin (HE) and immunohistochemical (IHC) staining

HE staining and IHC staining were performed as described previously [28]. For HE staining, mouse lung tissues were fixed in 4% paraformaldehyde. Fixed samples were embedded in paraffin, sectioned, and stained with hematoxylin and eosin. For IHC staining, formalin-fixed, paraffin-embedded tissues were sectioned, and slides were deparaffinized using xylenes (Fisher Scientific, USA). Tissue slides were rehydrated with a gradient of xylene and ethanol. Antigen retrieval was conducted using citric acid antigen retrieval buffer (Maixin, China), and endogenous peroxidase activity was blocked by hydrogen peroxidase for 20 min. Tissues were then incubated overnight at 4 °C with the following primary antibodies: Anti-AQP3 (2 ug/mL, ab125219, Abcam), Anti-CD163 (1:200, ab182422, Abcam), Anti-CD68 (1:500, ab303565, Abcam), Anti-IL-6 (1:200, GTX110527, GeneTex), Anti-Ki67 (1:200, ab16667, Abcam), Anti-GLUT1 (1:250, ab115730, Abcam), and Anti-LDHA (1:100, ab76315, Abcam). Subsequently, the slides were incubated with an HRP-conjugated secondary antibody (Zhongshan Golden Bridge, China) and visualized with a DAB kit. Finally, ImageJ IHC Profiler calculated LUAD AQP3 expression H-scores [29], and the median IHC score was used as the cut-off value to distinguish between high and low expression levels.

### Single-cell RNA sequencing (scRNA-seq)

Five cases of fresh LUAD samples and adjacent tissues were processed as follows. First, they were washed thrice with phosphate-buffered saline (PBS) and digested with an Enzymatic Tissue Dissociation Solution according to the manufacturer’s instructions [30]. Next, a single-cell suspension was prepared by resuspending the cells in PBS to a concentration of 1 × 10^5^ cells/mL, and this suspension was used to generate single-cell GEMs (gel beads in the emulsion) with the 10X Genomics Chromium system. scRNA-seq libraries were constructed using the 10X Genomics Chromium Single Cell 3’ Library & Gel Bead Kit v2 and sequenced with paired-end 150 reads on an Illumina HiSeq X10 instrument.

Raw gene expression matrices were generated using CellRanger (version 3.0.1) and processed using the Seurat R package (version 2.3.4). High-quality cells were selected based on the number of unique molecular identifiers (UMI) ( ≥ 400) and the proportion of intronic reads ( ≤ 40%). Data normalization was done with sctransform, while the dimensional reduction was done with uniform manifold approximation and projection (UMAP). The 12 major cell types were identified by combining an initial exploratory inspection of differentially expressed genes (DEGs) for each cluster with known marker genes and a literature study using the SingleR algorithm.

### ELISA

RAW264.7 cells were cultured according to the cell culture methods mentioned above. The culture medium derived from RAW264.7 cells was applied for incubation of LLC cells. Recombinant mouse IL-6 cytokines were detected in the RAW264.7-derived macrophages and the co-cultured LLC cells with or without AQP3 knockdown. Mouse IL-6 ELISA kits from Elabscience Biotechnology (Wuhan, China) were used for standard enzyme-linked immunosorbent assay (ELISA).

### Immunofluorescence staining tissue and cells

First, tissue slides or cells were fixed for 15 min in 4% paraformaldehyde. After washing thrice, fixed tissue slices or cells were permeabilized by 0.5% Triton X-100 for 5 min and blocked by goat serum for 1 h. Tissue or cells samples were then incubated with Anti-AQP3 (2 µg/mL, ab125219, Abcam), Anti-CD163 (1:50, sc-20066, Santa cruz), Anti-IL-6 (1:50, GTX110527, GeneTex), Anti-IL-6R (1:50, DF6466, Affinity), Anti- GLUT1 (1:200, ab115730, Abcam), Anti-LDHA (1 µg/mL, ab47010, Abcam) at 4 °C overnight, followed by the appropriate Alexa Fluor 488- or 594-conjugated secondary antibodies at 37 °C for 1 h. Tissue or cells were treated with DAPI for 15 min and observed by confocal or fluorescence microscopy (Nikon, Japan).

### Fluorescence flow cytometry

The cells, including THP-1, RAW264.7, M1/M2 type macrophages, and TAMs, were first blocked with 3% BSA and then incubated with a rabbit anti-CD163 antibody (dilution 1:50, ab182422) for 1 h at 4 °C. Subsequently, a goat anti-rabbit IgG (Alexa Fluor® 488) secondary antibody was applied for 30 min at room temperature. As a control, a rabbit IgG (1 μg/L × 10^6^ cells) isotype control antibody was also applied under the same conditions. Flow cytometry (BD Biosciences, USA) determined the percentage of the cells with specific staining and intensity.

The dissociated lung cells from mice were obtained using the mouse lung dissociation kit (Miltenyi Biotec, Germany) and gentleMACS dissociator (Miltenyi Biotec, Germany). To block non-specific binding, the cells were washed with flow staining buffer (PBS + 1% FBS) and then incubated with 10% goat serum at 4 °C for 15 min. After blocking, the cells were incubated with anti-CD11b APC antibody (17-0112-82, eBioscience), anti-F4/80 FITC antibody (11-4801-81, eBioscience), and anti-CD206 PE antibody (12-2061-80, eBioscience) at 4 °C for 20 min in the dark. The samples were rinsed with PBS by centrifugation at 400 g for 5 min at 4 °C. Finally, the cells were resuspended in Cell Dissociation Buffer (Invitrogen, USA) for flow cytometric analysis.

### Cell transfection and construction of stable cell lines

Small interfering RNA targeting AQP3 (si-AQP3) and plasmid of AQP3 overexpression (oe-AQP3) were synthesized by Hanheng Biotechnology (Shanghai, China). For transient transfection of si-AQP3, oe-AQP3, or corresponding controls into THP-1-derived or RAW264.7-derived M2 macrophages, Lipofectamine 3000 (Invitrogen, USA) reagent was applied according to the manufacturer’s manual.

For in vivo experiments, RAW264.7 macrophages with stable AQP3 knockdown were constructed according to the Stable Cell Line Construction Manual [31], followed by stably transfected cell lines acquired for further screening with puromycin (Beyotime, China).

### Cell proliferation assay

The EdU incorporation assay (Beyotime, China) and CCK-8 assay (Beyotime, China) were performed according to the manufacturer’s instructions to assess cell proliferation. Moreover, flow cytometry analysis was conducted to evaluate cell-cycle distribution. Cancer cells (A549 or LLC) were treated with M2 conditioned media, trypsinized, washed with PBS, and pelleted by low-speed centrifugation. Following overnight fixation with 75% ethanol at 4 °C, the cells were incubated with RNaseA at 37 °C for 30 min and propidium iodide (PI) at 4 °C for 30 min. Then the cells were analyzed by flow cytometry (BD Biosciences, USA).

### Transwell migration assay

A549 or LLC cells were cultured in the upper chamber (5 × 10^4^ cells/well) while THP-1-derived or RAW264.7-derived M2 were cultured in the lower chamber (2 × 10^5^ cells/well) using Boyden chambers system (8 μm pore size; BD Biosciences, USA) without Matrigel. After 48 h of incubation at 37 °C, migrating cells were fixed with methanol for 15 min and stained with crystal violet solution for 20 min. Finally, stained cells were photographed and counted under light microscopy.

### Cytokine-expression profile analysis

A human cytokine antibody array kit (AAM-CYT-1-2, Ray Biotech, USA) was used to detect 23 mouse cytokines simultaneously in culture supernatants from RAW264.7-derived M2 macrophages. The antibody array membranes were blocked for 30 min and incubated with 1 mL of undiluted culture supernatant at room temperature for 2 h. After washing, 1 mL of the prepared Biotinylated Antibody Cocktail was added overnight at 4 °C, followed by 2 h of incubation with 2 mL of 1X HRP-Streptavidin. After the final washes, the chemiluminescence membranes were photographed and digitized using ImageQuant LAS 4000 (GE Healthcare, UK).

### Glucose-6-phosphate dehydrogenase (G6PDH) and lactate dehydrogenase (LDH) activity assay

After treatment, cells were digested with 0.25% trypsin and washed with PBS. Using the BCA Protein Assay Kit (Solarbio, China), the total protein concentration was calculated and used to standardize the data. G6PDH activity was detected using G6PDH activity assay kit (Solarbio, Cat.# BC0260) following the manufacturer’s protocol. A lactate dehydrogenase activity assay kit (Solarbio, Cat.# BC0680) was used for LDH measurements. The G6PDH absorbance was measured at 340 nm, while the LDH absorbance was measured at 450 nm.

### Extracellular acidification rate

The XF glycolysis stress test kit (Seahorse Bioscience, USA) was employed to assess the extracellular acidification rate following the manufacturer’s instructions. A total of 5 × 10^4^ targeted cells per well were seeded into Seahorse plates, and 500 mL of medium was added, allowing for overnight incubation. The following day, the culture solution was removed, and each well was supplemented with XF Base Medium, with cells then incubated under starvation conditions for 2 h. Subsequently, the cells were subjected to treatment with glucose (10 nM), oligomycin (1 mM), and 2-deoxyglucose (50 nM) utilizing the XF24 Extracellular Flux Analyzer (Seahorse Bioscience in Billerica, MA, USA) for the measurement of extracellular acidification rate (ECAR).

### Animal experiments

All animal studies were conducted following the guidelines of the Animal Experiment Committee of Fujian Medical University. Male C57BL/6 J mice (4–6 weeks old, weighing 15–20 g) were obtained from the SLAC Laboratory Animal Company (Shanghai, China) and were used as the wild-type (WT) group. Aqp3 knockout (Aqp3^−/−^) male C57BL/6 J mice were generated by Cyagen Biosciences Company (Guangzhou, China) using CRISPR/Cas-mediated genome engineering. Guide RNAs were designed to target exon 1 and 6 of Aqp3. Cas9 mRNA and gRNA, generated by in vitro transcription, were injected into fertilized eggs for knockout mouse production. Founders were genotyped by PCR, followed by DNA sequencing analysis. The primers for genotyping Aqp3^−/−^ mice were 5’- CAACACTCACTCCCCTAAGAATCC-3’ (forward) and 5’- GCATCATTCAGCTTAGAAAACAGC-3’ (reverse). All animals were housed in a specific pathogen-free facility and maintained on a 12 h light/ 12 h dark schedule.

To induce the tumor formation, male C57BL/6 J mice (4–6 weeks, 15–20 g) were intraperitoneally injected with 1000 mg/kg urethane (Sigma-Aldrich, USA) twice a week for 15 weeks. At week 20, mice began to receive intraperitoneal injections of clodronate liposomes (4 mg per mouse, Liposoma BV, Netherlands), pathway agonists, or inhibitors once a week for five weeks. Mice were euthanized at time points up to 25 weeks after intervention.

To establish co-inoculated subcutaneous tumor allograft models, LLC cells (1 × 10^6^ mixed with 2 × 10^6^ macrophages in 200 µL PBS) were injected subcutaneously in the dorsal side of C57BL/6 mice as described previously [32, 33]. Mice were randomly chosen and assigned into two groups (8 mice per group) based on the difference of macrophages co-inoculated with LLC cells: (1) Control group (LLC cells + sh-NC-M2); (2) Treatment group (LLC cells + sh-Aqp3-M2). After implantation, tumor volumes and mouse weights were assessed every three days and estimated using the formula: volume = ab^2^/2, where a and b are the long and short diameters, respectively. On day 24 after tumor implantation, mice were executed at anesthesia.

### Statistical analyses

GraphPad Prism 8.4 (GraphPad Software, USA) and SPSS 23.0 (SPSS, USA) software were used to analyze the data. The relationship between the AQP3 expression and clinicopathological parameters was assessed by the χ2-test or Fisher’s exact. Kaplan–Meier plots were used for the overall survival (OS) and progression free survival (PFS) rates, and survival distributions were performed using the log-rank test. Student’s t-test and one-way analysis of variance (ANOVA) were employed to compare two or more groups. Statistical significance was set at *P* < 0.05.

## Results

### AQP3 was highly expressed in LUAD and correlated with tumor prognosis

**Fig. 1:**
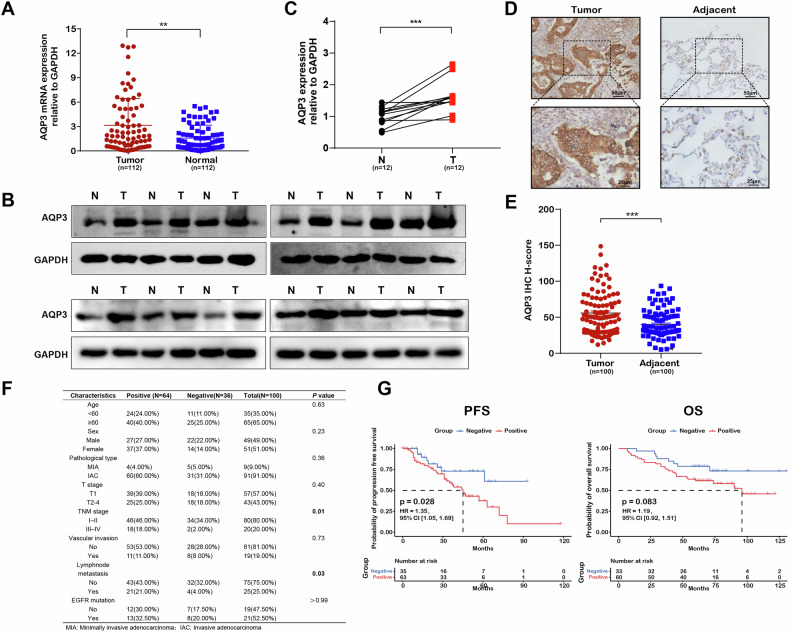
AQP3 expression in LUAD correlates with poorer outcome. **A** Relative mRNA expression levels of AQP3 in LUAD tissues and normal lung tissues determined by RT-qPCR. **B**, **C** Western blot analysis of AQP3 protein expression in stage I LUAD tissues and corresponding adjacent normal tissues (*n* = 12). **D**, **E** Representative images of AQP3 protein expression in stage I LUAD tissues and adjacent tissues detected by IHC, and corresponding H-score quantification using ImageJ IHC Profiler. **F** Correlation of AQP3 expression with clinicopathological characteristics of LUAD patients. **G** Kaplan-Meier survival analysis of progression-free survival (PFS) and overall survival (OS) in LUAD patients stratified by AQP3 protein expression. **P < 0.01, ***P < 0.001.

To explore the potential role of AQP3 in LUAD progression, we analyzed the expression and prognosis of AQP3 based on LUAD tissues. We used TCGA data to show that AQP3 was significantly elevated in different clinical stages of LUAD tissues compared to normal lung tissues, specifically in stage I LUAD [22]. We further validated the AQP3 mRNA expression in stage I LUAD by RT-qPCR. The results showed that the mRNA expression level of AQP3 was significantly higher in stage I LUAD than in normal tissues (*n* = 112, Fig. 1A). Subsequently, the protein expression of AQP3 in stage I LUAD patients was detected by Western blot. The results revealed that AQP3 was markedly upregulated in stage I LUAD tissues compared to adjacent normal lung samples (*n* = 12, Fig. 1B, C).

Additionally, IHC staining was further performed to confirm the AQP3 expression in LUAD tissues. H-score was used to quantify the staining by ImageJ IHC Profiler, and the results showed that the AQP3 expression level in cancer tissues was significantly higher than in adjacent tissues (*n* = 100, Fig. 1D, E). We identified AQP3 expressed mostly in the plasma membrane and a few in the cytoplasm. Then we further analyzed the association between AQP3 protein expression and clinicopathologic features in LUAD. Kaplan-Meier analysis demonstrated that the AQP3 expression was significantly correlated to TNM stage and lymph node-metastasis in LUAD patients (Fig. 1F). Importantly, elevated AQP3 expression showed a statistically significant influence on PFS and borderline significance on OS, suggesting a possible role in tumor growth and poor prognosis (Fig. 1G). Additionally, we observed a higher risk and poorer prognosis in LUAD patients those aged over 60 and in stage III-IV (Fig. S2A).

### AQP3 was overexpressed in TAMs of LUAD tissues based on single-cell sequencing analysis

**Fig. 2:**
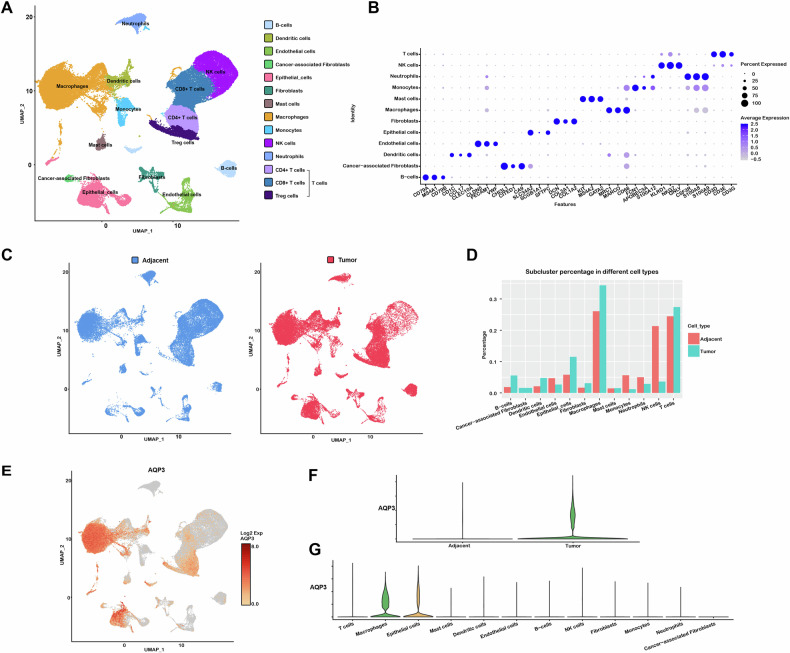
Overview of the clustering and annotation of the scRNA-seq for LUAD and adjacent lung tissues. **A** UMAP plot displaying 67,881 cells separated into 12 subtypes. **B** Dot plots showing markers of different cell populations. **C** UMAP showing the distribution of different sample types. **D** The bar chart displaying the expression differences among distinct cell populations in both LUAD and adjacent lung tissues. **E** Expression levels of AQP3 on UMAP plots. **F**, **G** Violin plot displaying the differential expression of AQP3 in distinct samples and subclusters.

And then, AQP3’s effects on the TME of LUAD were examined using scRNA-seq on fresh tumor samples and adjacent tissues from five treatment-naïve stage I LUAD patients. After initial quality control assessment and doublet removal, we obtained single-cell transcriptomes from a total of 67,881 cells, including 35,189 cells from LUAD samples and 32,692 cells from adjacent specimens (Fig. 2A). Unbiased clustering of the cells identified 12 main subclusters in parallel according to the typical type-specific gene markers [34], which were visualized by dot plots (Fig. 2B). Information on specific gene markers is presented in Table S2. Moreover, we visualized the expression levels of the top ten DEGs among each subcluster using a heatmap (Fig. S1). We also utilized UMAP and bar charts to show the cell distributions in LUAD and adjacent tissues and the proportion of each subcluster. (Fig. 2C, D).

**Fig. 3:**
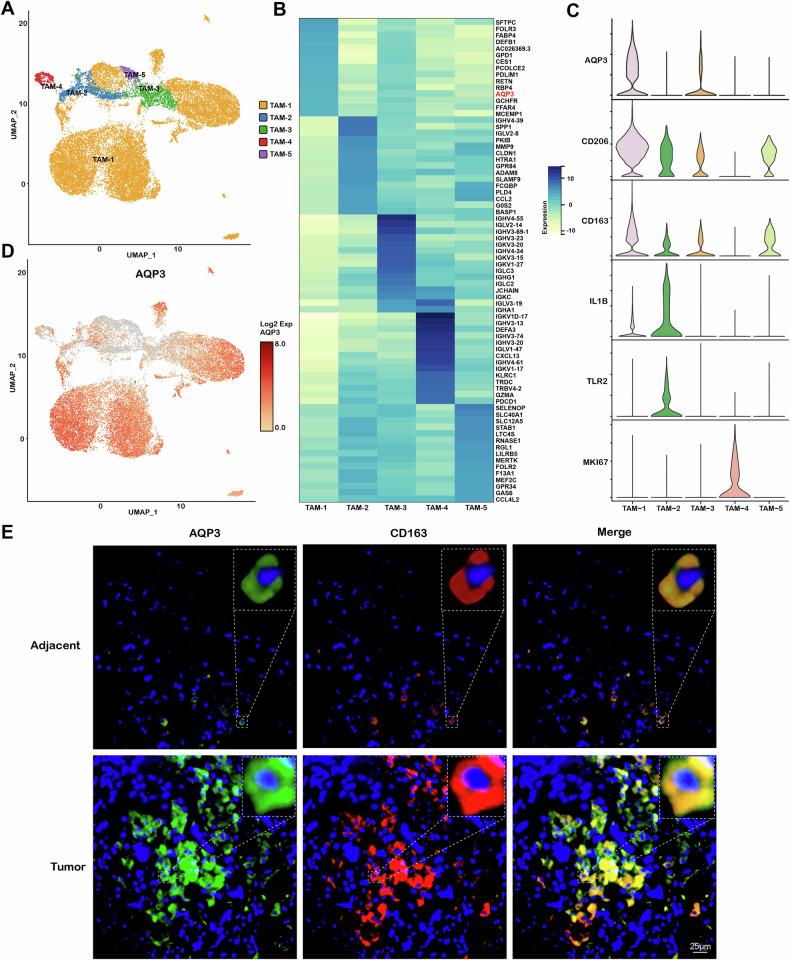
Detailed classification of macrophage subsets in LUAD. **A** UMAP plot displaying the five TAM subclusters identified in adjacent lung and LUAD samples. **B** Heatmap showing the top 15 highly expressed genes in each of the distinct macrophage subpopulations. **C** Violin plots displaying the expression levels of AQP3, CD206, CD163, IL-1B, TLR2, and MKI67 in different macrophage subtypes. **D** UMAP plot displaying the expression of AQP3 in each of the TAM subclusters. **E** Immunofluorescent staining showing the expression and co-localization of AQP3 and CD163 in adjacent lung and LUAD tissues.

Based on the scRNA-seq, we further investigated the expression distributions of AQP3 in LUAD. The results showed that the AQP3 expression in LUAD was higher than in adjacent samples (Fig. 2E, F). Previous studies revealed that AQP3 was predominantly expressed in epithelial cells in normal lung tissues [18]. However, our findings indicated that AQP3 was not only present in epithelial cells, but was also highly expressed in macrophages within LUAD samples (Fig. 2G). We then assessed the fine distributions of AQP3 in macrophages. As a type of TAMs, the macrophages furtherly were classified into five subclusters (TAM-1, TAM-2, TAM-3, TAM-4, and TAM-5) based on the DEGs by scRNA-seq (Fig. 3A, B). We found that M2 gene markers CD206 and CD163 were mostly expressed in TAM-1 cluster, while M1 gene markers IL-1B and TLR2 were mostly expressed in TAM-2, suggesting that TAM-1 may be M2-like and TAM-2 M1-like macrophages (Fig. 3C). Furthermore, we found that AQP3 was mainly expressed in TAM-1 subcluster (Fig. 3B–D). Therefore, we speculated that AQP3 might exert functions on LUAD by directly or indirectly regulating M2-like TAMs.

Subsequently, we conducted IHC staining employing CD68 and CD163 markers to evaluate the expression of AQP3 in TAMs within LUAD tissues. The results revealed a significant upregulation of AQP3 in TAMs of LUAD when compared to adjacent tissues (Fig. S2B). Similarly, double immunofluorescence assay with AQP3 and CD163 in primary LUAD was performed. As shown in Fig. 3E and Fig. S2C, AQP3, and CD163 were co-expressed in LUAD tissues, and the fluorescence intensity and infiltration density of co-expression were higher than in adjacent tissues, suggesting a potential role of AQP3 in M2 macrophages of LUAD microenvironment.

### Inhibition of AQP3 in M2 macrophages attenuated polarization process and reduced LUAD progression in vitro

**Fig. 4:**
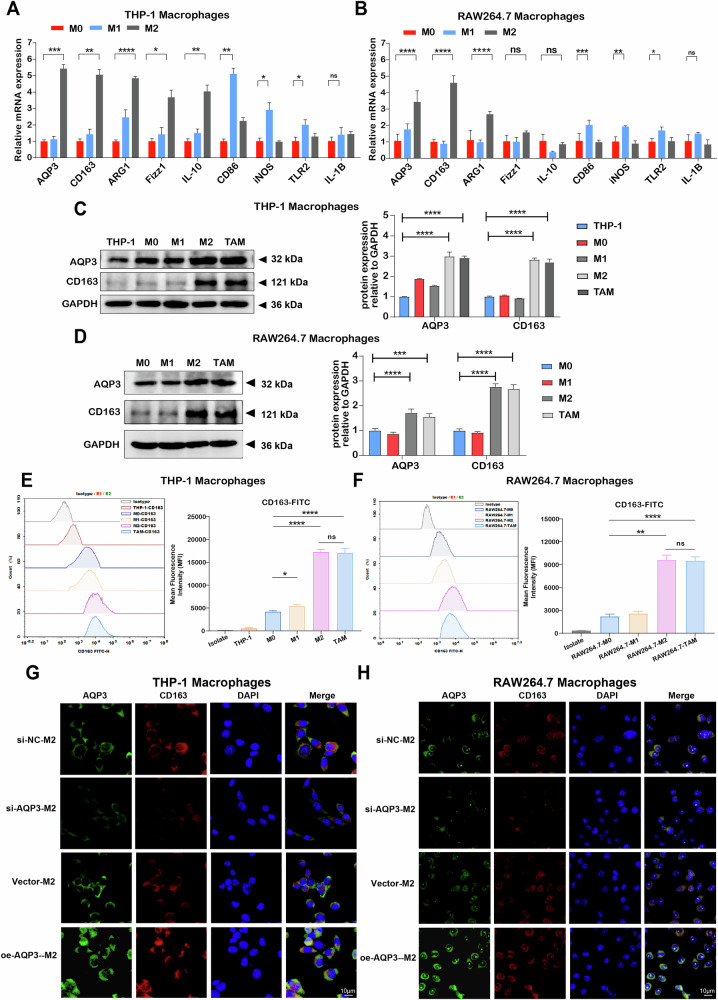
AQP3 was highly expressed in M2 macrophages and regulated the polarization of M2 macrophages. **A**, **B** RT-qPCR analysis of AQP3 and macrophage marker gene expression in different types of macrophages derived from THP-1 or RAW264.7 cells. **C**, **D** Western blot analysis of AQP3 and CD163 protein expression in THP-1 cells and different types of macrophages. **E**, **F** Flow cytometry analysis of CD163 expression in human or mouse-derived macrophages. **G**, **H** Immunofluorescence staining of AQP3 and CD163 expression in THP-1 or RAW264.7-derived macrophages after overexpression or knockdown of AQP3. *P < 0.05, **P < 0.01, ***P < 0.001, ****P < 0.0001.

To confirm the AQP3 expression in TAMs in vitro, we stimulated THP-1-derived and RAW264.7-derived macrophages to differentiate into M1 and M2 polarization, respectively. CD163 and ARG1 were highly expressed on THP-1-derived and RAW264.7-derived M2 macrophages, while CD86, iNOS, and TLR2 were highly expressed on THP-1-derived and RAW264.7-derived M1 macrophages. Importantly, our findings showed that AQP3 mRNA expression was significantly increased in THP-1-derived or RAW264.7-derived M2 macrophages compared to M0 and M1 macrophages (Fig. 4A, B).

Furthermore, the cancer cells were co-cultured with M0 macrophages to establish the TAMs model. Western blot analysis showed that AQP3 and CD163 had higher expressions in TAM and M2 macrophages than in THP-1-derived or RAW264.7-derived M0/M1 macrophages (Fig. 4C, D). Flow cytometry results showed similar results. The mean fluorescent intensity (MFI) of CD163 was significantly increased in TAMs and M2 macrophages compared with M0/M1 macrophages (Fig. 4E, F). Similarly, immunofluorescence staining also demonstrated that AQP3 and CD163 were highly expressed in THP-1-derived/RAW264.7-derived TAMs and M2 macrophages (Fig. S3A, B). Importantly, M2 macrophages and TAMs had identical protein expression levels and CD163 MFI, confirming that TAMs were M2 phenotype macrophages.

Moreover, loss-of-function and gain-of-function assays were performed to deeply validate the AQP3 roles in M2 macrophages in LUAD. The immunofluorescence staining and RT-qPCR confirmed that AQP3 was downregulated or elevated in M2 macrophages (Fig. 4G, H and S3C, D). We also found that the expression of M2 gene markers CD163, ARG1, and Fizz1 were significantly decreased in M2 macrophages after downregulating AQP3 while markedly increased in M2 macrophages after overexpressing AQP3 (Fig. S3C, D). Similar results were obtained from immunofluorescence staining (Fig. 4G, H), indicating that AQP3 inhibition could attenuate M2 macrophage polarization and AQP3 overexpression could promote polarization of M2 macrophages.

**Fig. 5:**
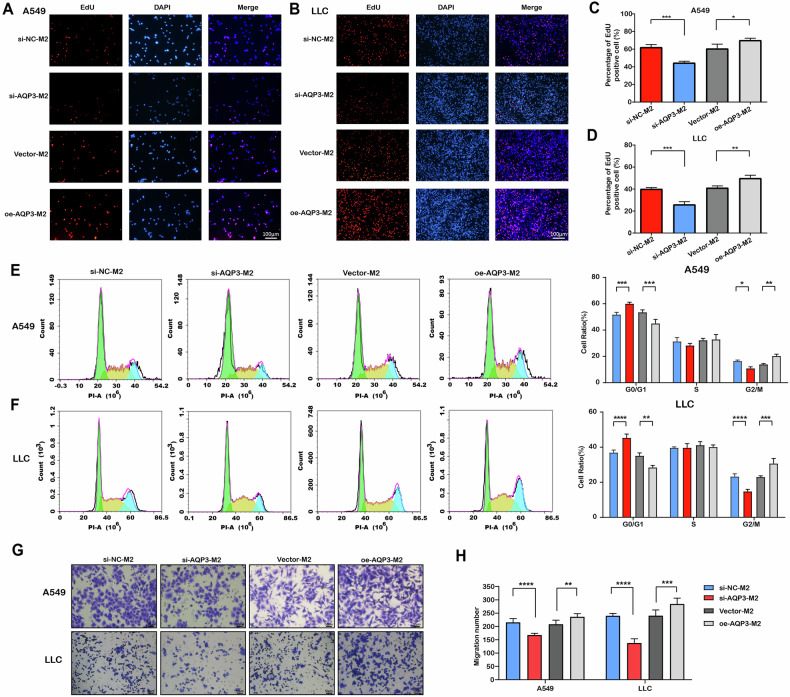
AQP3-mediated M2 polarization further affected the proliferation and migration of cancer cells. **A**−**D** Fluorescence images and bar graphs showing the EdU assay results after co-culture of A549 and LLC cells with human and mouse-derived M2 macrophages with knockdown or overexpression of AQP3. **E**, **F** Peak graphs and bar graphs displaying the flow cytometry analysis results after co-culture of A549 and LLC cells with AQP3-modified M2 macrophages. **G**, **H** Transwell assay results of A549 and LLC cells co-cultured with human and mouse-derived M2 macrophages with knockdown or overexpression of AQP3. *P < 0.05, **P < 0.01, ***P < 0.001, ****P < 0.0001.

Co-culture experiments were performed to further explore the possible effect exerted by AQP3 on the tumor in vitro. After inhibiting AQP3 expression in M2 macrophages and co-cultivating with cancer cells, we found that M2 macrophages with AQP3 knockdown could significantly reduce the proliferation, cell cycle, and migration of tumor cells compared with the control group by CCK-8, EdU, flow cytometry, and transwell assay (Fig. 5 and S4). In contrast, AQP3 overexpression in M2 macrophages could promote tumor cells proliferation and migration (Fig. 5 and S4).

### AQP3 regulated macrophage polarization dependent on the PPAR-γ/NF-κB axis

**Fig. 6:**
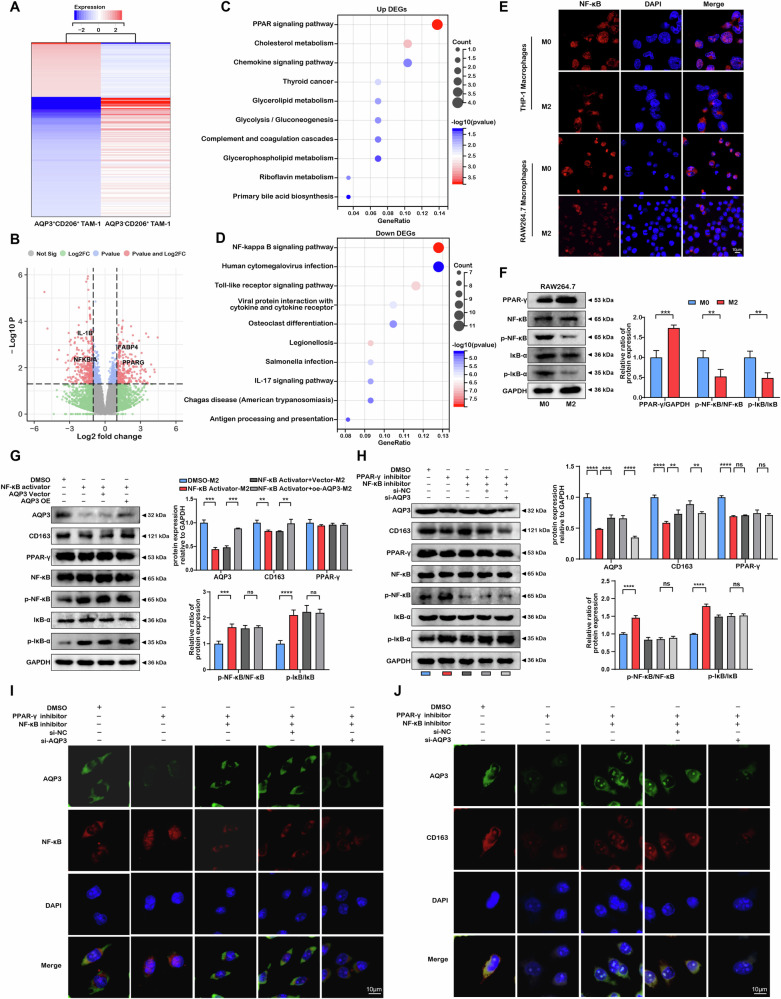
AQP3-mediated M2 macrophage polarization via PPAR-γ/NF-κB axis. **A**, **B** Heatmap and volcano plots displaying DEGs between AQP3^+^CD206^+^ TAM-1 and AQP3^-^CD206^+^ TAM-1 clusters via scRNA-seq. **C** KEGG plot showing pathway enrichment of upregulated DEGs in AQP3^+/-^ CD206^+^ TAM-1 clusters. **D** KEGG plot showing pathway enrichment of downregulated DEGs in AQP3^+/-^ CD206^+^ TAM-1 clusters. **E** Immunofluorescence double staining to determine NF-κB nuclear translocation in different types of macrophages. **F** Western blot determination of the protein expressions of PPAR-γ, NF-κB, p-NF-κB, IκB-α, and p-IκB-α in THP-1 or RAW264.7-derived macrophages. **G** Western blot detection of protein expression of PPAR-γ, NF-κB, p-NF-κB, IκB-α, and p-IκB-α in mouse-derived macrophages under NF-κB activator or AQP3 overexpression intervention. **H** Western blot analysis of the protein expressions of PPAR-γ, NF-κB, p-NF-κB, IκB-α, and p-IκB-α in RAW264.7-derived macrophages after intervention of PPAR-γ inhibitor, NF-κB inhibitor, or AQP3 knockdown. **I**, **J** Immunofluorescence staining of AQP3, NF-κB, and CD163 after intervention of PPAR-γ inhibitor, NF-κB inhibitor, or AQP3 knockdown. **P < 0.01, ***P < 0.001, ****P < 0.0001.

Subsequently, we deeply explored the potential pathways by analyzing DEGs between AQP3^+^CD206^+^ TAM-1 and AQP3^-^CD206^+^ TAM-1 clusters based on scRNA-seq (Fig. 6A). We noticed that PPARG was one of the significant upregulated genes and NFKBIA was one of significant downregulated genes (Fig. 6B). Further KEGG enrichment analysis indicated that the up-regulated DEGs were significantly enriched on PPAR signaling pathway, while the down-regulated genes were mostly enriched on NF-κB signaling pathway (Fig. 6C, D).

To confirm that AQP3 was indeed associated with PPAR pathway and NF-κB pathway in M2 macrophages. We first examined the relationship between M2 macrophages and NF-κB pathway by immunofluorescent staining and Western blot. As shown in Fig. 6E, there was a significant nuclear localization of NF-κB in THP-1-derived or RAW264.7-derived M0 macrophages, but not in M2 macrophages. Western blot results also showed that p-NF-κB and p-IκB-α were highly expressed in M0 macrophages than those in M2 macrophages (Fig. 6F). Next, we investigated the roles of NF-κB pathway in AQP3-induced M2 macrophages using rescue experiments. NF-κB activation significantly inhibited the protein expression of AQP3 and CD163, while AQP3 overexpression could not change the protein expression levels of NF-κB pathway (Fig. 6G). Similar findings were obtained from RT-qPCR (Fig. S5A). These findings suggested that NF-κB pathway was a key upstream regulator for AQP3.

Several studies identified PPAR-γ as a key negative regulator for NF-κB [35, 36]. Our study found that PPAR-γ was significantly upregulated in M2 macrophages. Importantly, PPAR-γ inhibition reduced the protein expression levels of AQP3, CD163, p-NF-κB, and p-IκB-α, while NF-kB inactivation and AQP3 knockdown could not change the PPAR-γ protein expression (Fig. 6H). RT-qPCR analysis (Fig. S5B) and immunofluorescence assay (Fig. 6I, J) demonstrated similar results. These findings indicated that the PPAR-γ/NF-κB axis was a key upstream pathway of AQP3 for M2 macrophages polarization.

### IL-6 was the key downstream molecule of AQP3-mediated tumor progression

**Fig. 7:**
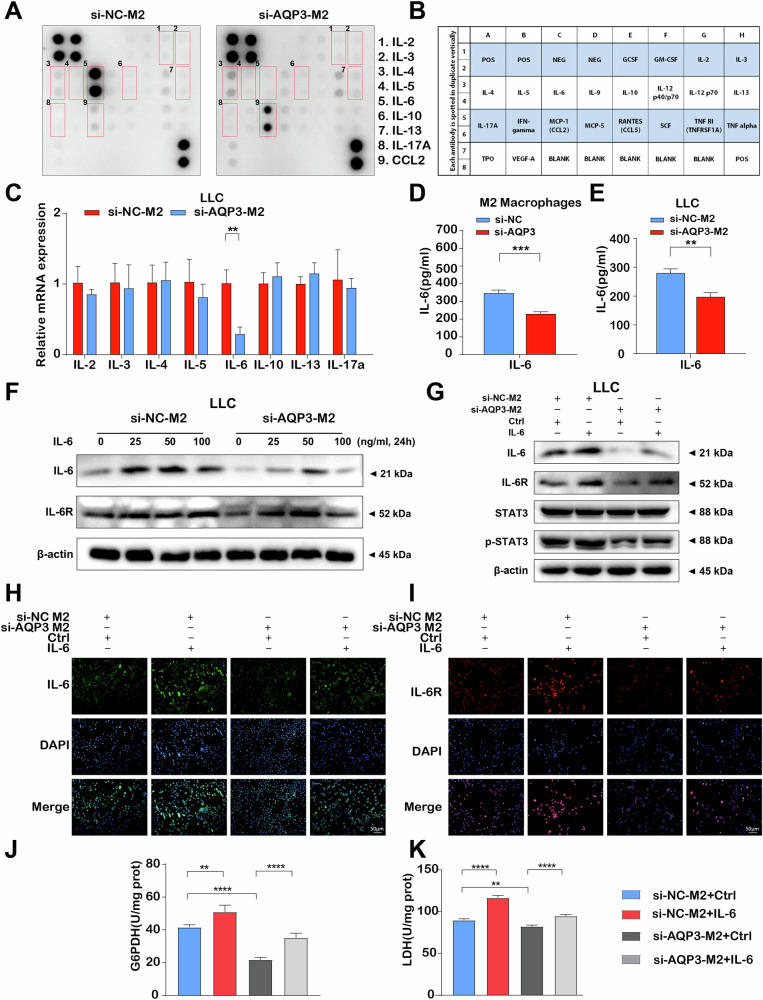
IL-6 was the key downstream molecule of AQP3-mediated tumor progression. **A** Cytokine antibody microarray image displaying the expression of cytokines in the supernatant of mouse-derived macrophages with AQP3 knockdown. **B** The cytokine antibody chip membrane showing different spots corresponding to different cytokines. **C** RT-qPCR analysis of cytokine expression in LLC cells after co-culture with macrophages. **D**, **E** ELISA analysis of IL-6 expression in AQP3-knockdown M2 macrophages and co-cultured LLC cells. **F** Western blot analysis of IL-6 and IL-6R protein expressions under different concentrations of IL-6 stimulation. **G** Western blot analysis of IL-6, IL-6R, STAT3 and p-STAT3 protein expressions in LLC cells co-cultured with si-AQP3 and IL-6 intervention. **H**, **I** Immunofluorescence staining of IL-6 and IL-6R fluorescence intensity in LLC cells co-cultured with si-AQP3 and IL-6 intervention. **J**, **K** G6PDH and LDH activity assays detecting glucose metabolism in LLC cells co-cultured with si-AQP3 and IL-6 intervention. POS: positive controls; NEG: negative controls. **P < 0.01, ***P < 0.001, ****P < 0.0001.

However, the downstream mechanism of AQP3-mediated tumor suppression is relatively unknown. We analyzed cytokine-expression profiles in the supernatants of AQP3-mediated M2 macrophages. Representative images of the cytokine antibody array are shown in Fig. 7A, B, in the supernatant of the si-AQP3-M2 macrophages group, IL-6 expression was strongly decreased. Among all cytokines, only IL-6 mRNA expression was significantly downregulated in tumor cells co-cultured with AQP3-inhibited M2 macrophages (Fig. 7C). Similarly, ELISA assay also substantiated that IL-6 was not only reduced in AQP3 knockdown M2 macrophages, but also in LLC co-cultured with AQP3-inhibited M2 macrophages (Fig. 7D, E).

It is well known that glucose metabolism plays a critical role in lung adenocarcinoma by promoting cell proliferation, invasion, and metastasis through enhanced glycolytic pathways known as the Warburg effect [37, 38]. Previous research has concluded that IL-6 was closely correlated with the occurrence and development of tumors and affected tumorigenesis and glucose metabolism through the IL-6R/STAT3 axis [39, 40]. To further elucidate the role of AQP3-mediated IL-6 in LUAD progression, we first determined and chose 50 ng/mL as the optimal concentration of IL-6 for rescue assay by Western blot (Fig. 7F). Compared to the control group, the protein expressions of IL-6, IL-6R, and p-STAT3 were decreased in LLC co-cultured with AQP3-inhibited M2 group. In contrast, they were distinctly increased in co-cultured LLC after stimulating with IL-6 (Fig. 7G). Similar findings were presented in immunofluorescent staining of IL-6 and IL-6R (Fig. 7H, I). Moreover, we detected the proliferation, migration, and glucose metabolism of co-cultured LLC cells. CCK-8, EdU, and transwell assays showed that IL-6 could reverse the inhibitory effects of proliferation and migration of tumor cells owing to AQP3 knockdown in co-cultured model (Fig. S6A–E). Similarly, fluorescence detection of GLUT1 and LDHA (Fig. S6F, G), ECAR assay (Fig S6H), G6PDH, and LDH activities assays (Fig. 7J, K) demonstrated that IL-6 stimulation also reversed the glucometabolic changes in AQP3-inhibited co-cultured cells. Thus, these results suggested that AQP3 mediated the secretion of IL-6 in M2 macrophages and further affected the role of IL-6 in tumor initiation and progression.

### Macrophages were an important factor affecting tumor progression in LUAD microenvironment

**Fig. 8:**
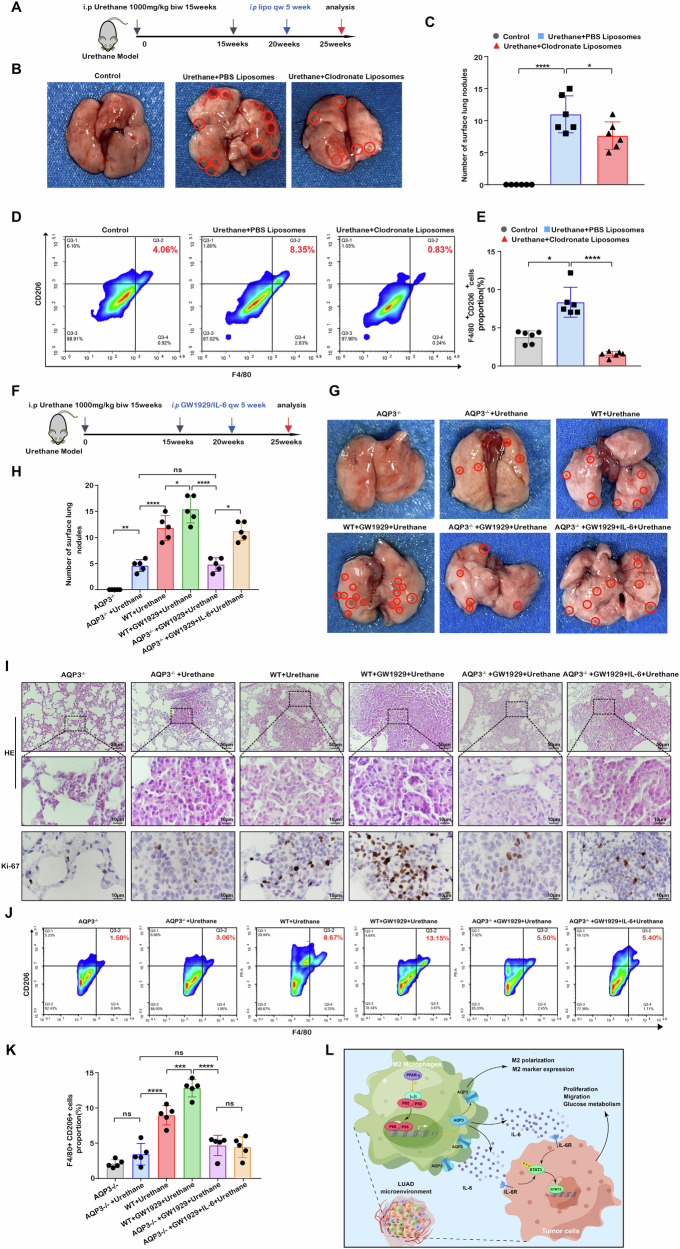
The effect of AQP3-mediated macrophages on the urethane mouse model. **A** The method, concentration, and duration of urathane and clodronate liposomes intervention in C57BL/6 J mice. **B** Representative images of surface lung nodules in C57BL/6 J mice following 25 weeks of intervention. **C** Bar graph showing the number of surface lung nodules in different groups. **D**, **E** Flow cytometry analysis of the proportion of F4/80^+^CD206^+^ cells in different groups. **F** The method, concentration, and duration of urethane, GW1929, and IL-6 intervention in C57BL/6 J mice. **G** Representative images of surface lung nodules in C57BL/6 J mice after intervention with urethane, GW1929, and IL-6. **H** Bar graph showing the analysis of surface lung nodules in C57BL/6 J mice after intervention with urethane, GW1929, and IL-6. **I** HE staining and Ki-67 staining of lung nodule lesions in C57BL/6 J mice with different interventions. **J**, **K** Flow cytometry analysis of the proportion of F4/80^+^CD206^+^ cells in C57BL/6 J mice with urethane, GW1929, and IL-6 intervention. **L** The schematic diagram illustrates the proposed mechanism of AQP3 in the tumor microenvironment of LUAD. biw = twice a week; i.p. = intraperitoneal injections; Lipo = clodronate liposomes; qw = once a week; GW1929 = PPAR-γ activator. *P < 0.05, ***P < 0.001, ****P < 0.0001.

To clarify whether TAMs and macrophage polarization affect tumor development in vivo, we first performed an intervention of clodronate liposomes to deplete macrophages in a urethane-induced lung cancer model. All mice were sacrificed after model establishment and drug intervention (Fig. 8A). Compared to untreated mice, urethane-induced mice had large grey nodules and dark-red congested regions on their lungs (Fig. 8B). Quantitative analysis showed that the number of surface lung nodules was lower in urethane + clodronate liposomes group than in urethane + PBS liposomes group (Fig. 8C). HE staining demonstrated that pulmonary nodules had large and slightly aberrant nuclei with a high nucleus-to-cytoplasm ratio. Still, no morphological differences were seen between urethane-induced clodronate liposomes and PBS liposomes group (Fig. S7A). Additionally, IHC staining demonstrated the reduced protein levels of Ki-67 in the urethane-induced clodronate liposomes group (Fig. S7A). Furthermore, the proportion of F4/80^+^CD206^+^ macrophages was significantly decreased after the treatment of clodronate liposomes in the urethane-induced group (Fig. 8D, E). These results identified that clodronate liposomes could reduce the M2 macrophage ratio and suppress lung tumor progression.

### The roles of AQP3 in M2 macrophages polarization and tumorigenesis and progression in vivo

We created two types of AQP3-associated tumor models to explore the role of AQP3 on LUAD in vivo. In the co-inoculated subcutaneous tumor allograft model, the tumor volume and weight of the sh-Aqp3-M2 + LLC cell group were smaller than in the sh-NC-M2 + LLC cell group (Fig. S7B–D). HE staining of tumor sections showed an abnormal nuclear-cytoplasmic ratio between the two groups, but there was no significant difference (Fig. S7E). Moreover, the results of IHC showed that the expression of Ki-67 was significantly weakened in the sh-Aqp3-M2 + LLC cell group compared with the sh-NC-M2 + LLC cell group (Fig. S7F) and downregulated immunofluorescent intensity and IHC staining intensity of CD163 (Fig. S7G–I). Sh-Aqp3-M2 + LLC cells had considerably lower G6PDH and LDH activity than sh-NC-M2 + LLC cells (Fig. S7J, K).

In urethane-induced Aqp3 knockout mice (Fig. 8F), we observed a significant increase in pulmonary surface nodules in the WT + urethane group compared with the Aqp3^−/−^ urethane group. Moreover, the number of surface lung nodules was significantly higher in the WT + GW1929 + urethane group, demonstrating that Aqp3 deletion might decrease tumor growth while PPAR-γ agonists can promote tumor progression. Furthermore, we observed that the number of nodules on the lung surface in the Aqp3^−/−^ + GW1929 + urethane group was not significantly different from that in the Aqp3^−/−^ + urethane group, but it increased in the Aqp3^−/−^ + GW1929 + IL-6 + urethane group (Fig. 8G, H). The histological examination of pulmonary nodules using HE and Ki-67 staining revealed comparable findings (Fig. 8I).

Subsequently, we investigated the effect of Aqp3 on M2 macrophage polarization and glucose metabolism in lung tissues of mice. Our findings showed that Aqp3 knockout significantly reduced the proportion of F4/80^+^CD206^+^ cells compared to the WT + urethane group in lung tissue of mice. However, there were no significant changes in the proportion of F4/80^+^CD206^+^ cells stimulated by GW1929 and IL-6 in Aqp3 knockout mice (Fig. 8J, K). Our IHC analysis revealed that Aqp3 expression was negative in all knockout mice, with the most significant staining observed in wild-type mice, especially in the WT + GW1929 + urethane group. CD163 staining was denser in wild-type mice than in Aqp3 knockout mice (Fig. S8A). IL-6, GLUT1, and LDHA immunohistochemical staining increased in wild-type and IL-6-stimulated Aqp3 knockout mice, but the changes were insignificant (Fig. S8A). Our G6PDH and LDH activity detection experiments confirmed that the expression of G6PDH and LDH was significantly higher in the WT + urethane group than in the Aqp3^−/−^ + urethane group, and the expression in the WT + GW1929 + urethane group was the most significant. The G6PDH and LDH expression in the Aqp3^−/−^ + GW1929 + urethane group was not significantly different from the Aqp3^−/−^ + urethane group but significantly increased in the Aqp3^−/−^ + GW1929 + IL-6 + urethane group (Fig. S8B, C). These findings showed that Aqp3 was essential for M2 macrophage polarization, tumor development, glucose metabolism, and upstream and downstream pathways.

## Discussion

Numerous studies have established the considerable intra-tumor heterogeneity of LUAD, posing a formidable obstacle to developing successful treatment regimens. As an important member of the aquaporin family, the critical role of AQP3 in tumors has been increasingly recognized. AQP3, closely associated with clinical prognosis, was considerably enriched in TAM subsets of the microenvironment of LUAD, as shown by scRNA-seq, in vivo and in vitro investigations. Our results demonstrated that AQP3 relied on the PPAR-γ/NF-κB axis to regulate tumor occurrence and progression by mediating M2 macrophage polarization and IL-6 secretion (Fig. 8L). These findings provide a new research basis and idea for early diagnosis and drug target therapy of lung cancer.

scRNA-seq is used to research the tumor microenvironment due to its ability to detect rare and complex cell types, elucidate regulatory links between genes and cell subsets, and facilitate the development of targeted therapeutics and immunotherapies [16, 41]. In our study, we performed a comprehensive scRNA-seq analysis of stage I LUAD tissue, applying UMAP visualization and identifying representative marker genes to classify 12 distinct cell subgroups in both LUAD and adjacent tissues. These subpopulations included immune cells like macrophages, B cells, and T cells in the tumor microenvironment. Besides epithelial cells, AQP3 was significantly enriched in macrophage subpopulations. To gain more insights into this finding, we investigated the macrophages subclusters and found that AQP3 was most significantly expressed in TAM-1 subpopulations. TAM-1 is a subset of M2-like macrophages, suggesting that AQP3 might be closely related to M2-type macrophages in the LUAD microenvironment.

Prior research reveals that AQP3 regulates macrophage phagocytosis and migration by modulating water and glycerol transport [42]. AQP3 has also been shown to regulate H_2_O_2_ transport and activate macrophages to participate in liver injury development, making it a potential therapeutic target [43]. However, the relationship between AQP3 and macrophages in lung cancer has not been explored. In this study, we investigated the expression and function of AQP3 in macrophages in vitro. Our data demonstrated a substantial increase of AQP3 in M2 macrophages, with AQP3 mediating the polarization of M2 macrophages. Knocking down AQP3 in M2 macrophages inhibited cell cycle progression, proliferation, and migration of lung cancer cells, while AQP3 overexpression promoted these cell activities in M2 macrophages. These results are consistent with previous findings that AQP3 inhibits the proliferation and migration of lung cancer cell lines and that M2 macrophages are vital in regulating lung cancer cell proliferation [14, 44, 45]. Therefore, we believe that the AQP3 role in lung adenocarcinoma tissue is not limited to its direct effect on cancer cells but rather its ability to indirectly mediate the polarization of M2 macrophages, which can affect the occurrence and progression of lung cancer.

PPAR-γ is a critical transcription factor for macrophage activation and polarization, while NF-κB regulates the expression of inflammatory molecules and polarization of M2 macrophages [46–48]. Our scRNA-seq analysis of AQP3^+^ CD206^+^ TAM-1 and AQP3^–^ CD206^+^ TAM-1 groups revealed that PPAR signaling pathways and NF-κB pathways were enriched in upregulated and downregulated DEGs, respectively. AQP3 has a potential interaction relationship with PPAR-γ and NF-κB in the microenvironment of lung adenocarcinoma. Previous research indicated that NF-κB was a key transcription factor downstream of PPAR-γ, and PPAR-γ may block the activation of inflammatory genes by negatively regulating the NF-κB signaling pathway in macrophages [49].

Moreover, previous studies have shown that PPAR-γ agonists could promote the AQP3 gene expression in epidermal tissue and hepatic stellate cells [50, 51]. Despite the inhibitory role that PPAR-γ plays in various tumors [52–54], the regulatory relationship between PPAR-γ and AQP3 has not been reported in LUAD. Therefore, we hypothesized that the PPAR-γ/NF-κB signaling axis modulated AQP3 expression and influenced M2 macrophage polarization. In the recovery experiment, we found that PPAR-γ inhibited the AQP3 expression, which weakened the degree of M2 macrophage polarization, and negatively regulated the expression of NF-κB. Meanwhile, AQP3 knockdown failed to induce the PPAR-γ and NF-κB expression, which confirmed that the PPAR-γ/NF-κB axis was the upstream regulatory pathway of AQP3.

IL-6 is an important inflammatory cytokine involved in various inflammatory processes and inflammation-related tumors [39]. AQP3 regulates IL-6 expression in different contexts, including during macrophages priming and Helicobacter pylori infection. Loss of AQP3 might lead to reduced expression of inflammatory factors like IL-6 and TNF-α, and AQP3 inhibition could decrease the production of IL-6, IL-1β, and TNF-α, impairing intestinal bacteria clearance and contributing to gastric cancer progression [55–57]. In this study, only IL-6 exhibited a favorable effect on si-AQP3-M2 macrophages, despite several cytokines in the supernatant of M2 macrophages following AQP3 intervention. The significant decrease in the supernatant of IL-6 suggested that it was a potential downstream molecule of AQP3-mediated polarization of M2 macrophages. Subsequent experiments showed that adding IL-6 intervention to AQP3-knockdown M2 macrophages significantly increased the expression of IL-6, IL-6R, and p-STAT3, promoting the proliferation and migration of LLC cells with enhanced glucose metabolism. Consistent with earlier research, these findings show that IL-6 regulates tumor formation and glucose metabolism via the IL-6R/STAT3 axis [40, 58]. Therefore, we concluded that IL-6 might serve as a key downstream molecule of AQP3, exerting regulatory effects on the progression and glucose metabolism of lung cancer cells.

Urethane-induced tumors are a well-established animal model for investigating LUAD due to their standardized and highly reproducible tumor growth [59, 60]. This study investigated the macrophage polarization role in constructing the urethane-induced lung adenocarcinoma model. By using clodronate sodium liposomes to clear macrophages, we found that macrophages were key factors in the tumor microenvironment essential for tumor formation and progression, consistent with previous studies on the influence of macrophages on tumorigenesis [61–63]. Furthermore, our findings revealed that subcutaneous tumors in the sh-Aqp3-M2 + LLC cell group had significantly decreased weight, proliferation level, M2 polarization, and glucose metabolism compared to those in the control group. The findings from both in vivo and in vitro suggest that AQP3 regulates the M2 macrophage polarization in the tumor microenvironment and affects LUAD development.

Additionally, the knockout of Aqp3 has been found to prevent skin cancer progression in mice exposed to tumor inducers, indicating that AQP3 is a key molecule in skin cancer progression [64, 65]. The Aqp3 knockout mice model displayed slower urethane-induced lung adenocarcinoma formation, reduced tumor proliferative activity and glucose metabolism, and weaker M2 macrophage polarization. However, PPAR-γ agonists did not affect tumorigenesis in the Aqp3 knockout mice, but IL-6 secretion increased the number of nodules and tumor proliferation. The results suggest that AQP3 plays a key role in tumor development by mediating M2 polarization and IL-6 secretion.

However, there are some deficiencies in the present research. First, the number of LUAD patients and sample size of clinical specimens were not large enough. Second, upstream and downstream pathways of AQP3 must be validated using protein-protein and RNA interaction assays. Finally, the complete knockout mice model may impact the expression of gene regulatory network. Future studies using a conditional knockout mice model will be essential to finally demonstrate the role of AQP3 in tumor progression.

## Conclusion

In summary, the present study demonstrates AQP3 regulates tumor cell proliferation and migration by interfering with M2 macrophage polarization. The PPAR-γ/NF-κB axis is the upstream signal pathway of AQP3-mediated M2 macrophage polarization, while IL-6 is the key downstream molecule of AQP3-mediated tumor progression. These discoveries give a new theoretical and experimental foundation for lung cancer early detection and therapeutic research.

### Supplementary information

Supplemental Data

Western blot original image

## Data Availability

The data that support the findings of this study are available from the corresponding authors upon reasonable request.
