# Supplementary material for: PPAR-γ/NF-kB/AQP3 axis in M2 macrophage orchestrates lung adenocarcinoma progression by upregulating IL-6
Source: Cell Death Dis. 2024 Jul 26;15(7):532. doi: 10.1038/s41419-024-06919-9 (PMC11282095; doi:10.1038/s41419-024-06919-9)
Supplement: Supplementary file 1 — Supplemental Data [file 41419_2024_6919_MOESM1_ESM.docx]

***Supplementary Material***

| **Supplementary Tables**  Table S1 Primer sequence information | | | |
| --- | --- | --- | --- |
| gene | Forward sequence（5’-3’） | Reverse sequence（5’-3’） | |
| hsa-AQP3 | CTACCTACCCCTCTGGACACTTGG | | CACGAAGACACCCGCAATGGAG |
| mus-AQP3 | CCTTGGCATCTTGGTGGCT | | AGGAAGCACATTGCGAAGGT |
| hsa-CD163 | TTCCTGTTCTGGACGTGTGG | | AGCTGGACCACAGCCAAGTT |
| mus-CD163 | CTGGCGGGTGGTGAAAACA | | CAGCCGTTACTGCACACTG |
| hsa-ARG1 | CCTTTGCTGACATCCCTAAT | | GATTCTTCCGTTCTTCTTGACT |
| mus-ARG1 | CTCCAAGCCAAAGTCCTTAGAG | | GGAGCTGTCATTAGGGACATCA |
| hsa-Fizz1 | CCGTCCTCTTGCCTCCTTC | | CTTTTGACACTAGCACACGAGA |
| mus-Fizz1 | CTG​CTA​CTG​GGT​GTG​CTT​GT | | GCA​GTG​GTC​CAG​TCA​ACG​AG |
| hsa-IL-10 | GGCACCCAGTCTGAGAACAG | | TGGCAACCCAGGTAACCCTTA |
| mus-IL-10 | GGGTTGCCAAGCCTTATCGGAAAT | | CCTTGATTTCTGGGCCATGCTTCT |
| hsa-CD86 | CTGCTCATCTATACACGGTTACC | | GGAAACGTCGTACAGTTCTGTG |
| mus-CD86 | TCAATGGGACTGCATATCTGCC | | GCCAAAATACTACCAGCTCACT |
| hsa-iNOS | GAGCCAGGCCACCTCTATGT | | GTCCTCGACCTGCTCCTCAT |
| mus-iNOS | GGC​AGC​CTG​TGA​GAC​CTT​TG | | GCA​TTG​GAA​GTG​AAG​CGT​TTC |
| hsa-TLR2 | ATCCTCCAATCAGGCTTCTCT | | GGACAGGTCAAGGCTTTTTACA |
| mus-TLR2 | CTCTTCAGCAAACGCTGTTCT | | GGCGTCTCCCTCTATTGTATTG |
| hsa-IL-1β | AAACGAATGAAGTGCTCCTTCAGC | | ACCTCGTTGTTCACCACAAGAGGT |
| mus-IL-1β | GCAACTGTTCCTGAACTCAACT | | ATCTTTTGGGGTCCGTCAACT |
| hsa-GAPDH | CTCCTGCACCACCAACTGCTTAG | | GACGCCTGCTTCACCACCTTC |
| mus-GAPDH | CTT​CAC​CAC​CAT​GGA​GAA​GGC | | GGC​ATG​GAC​TGT​GGT​CAT​GAG |

| Table S2 Information of cell specific gene markers | |
| --- | --- |
| cell type | marker genes |
| B cells | CD79A, MS4A1, CD79B |
| Dendritic cells | CD1C, CCL17, CLEC10A |
| Endothelial cells | CLDN5, RAMP2, VWF |
| Cancer-associated fibroblasts | CHI3L1, CITED1, CA9 |
| Epithelial cells | SLC34A2, SCGB1A1, SFTPC |
| Fibroblasts | DCN, COL3A1, COL1A2 |
| Mast cells | KIT, MS4A2, GATA2 |
| Macrophages | MRC1, MARCO, CD68 |
| Monocytes | FCN1, APOBEC3A, S100A12 |
| NK cells | KLRD1, NKG7, GNLY |
| Neutrophils | CSF3R, S100A8, S100A9 |
| T cells | CD3D, CD3E, CD3G |

**Supplementary Figures**


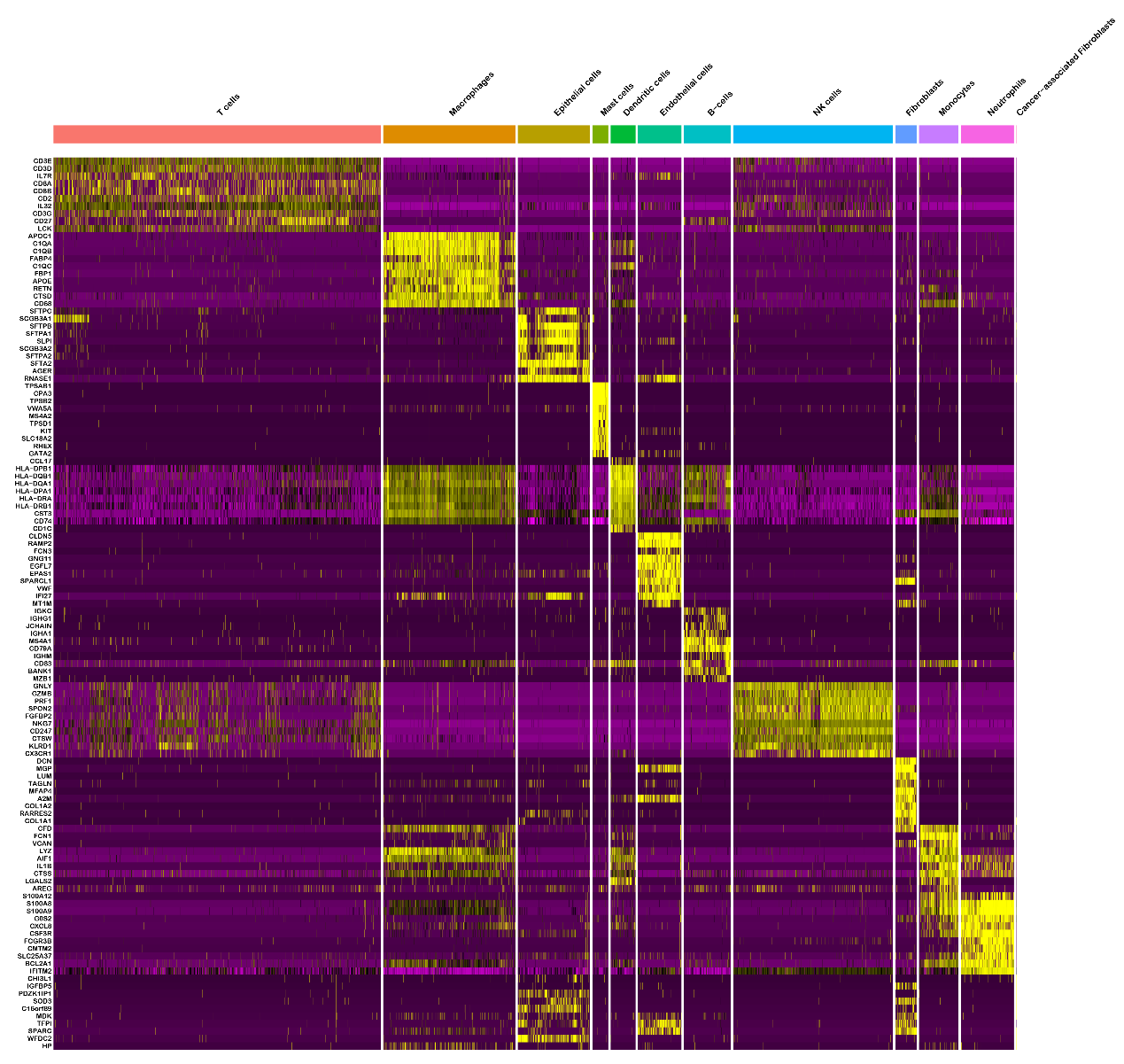


**Fig. S1 Heatmap displayed the expression levels of the top ten differentially expressed genes (DEGs) between each subcluster.**


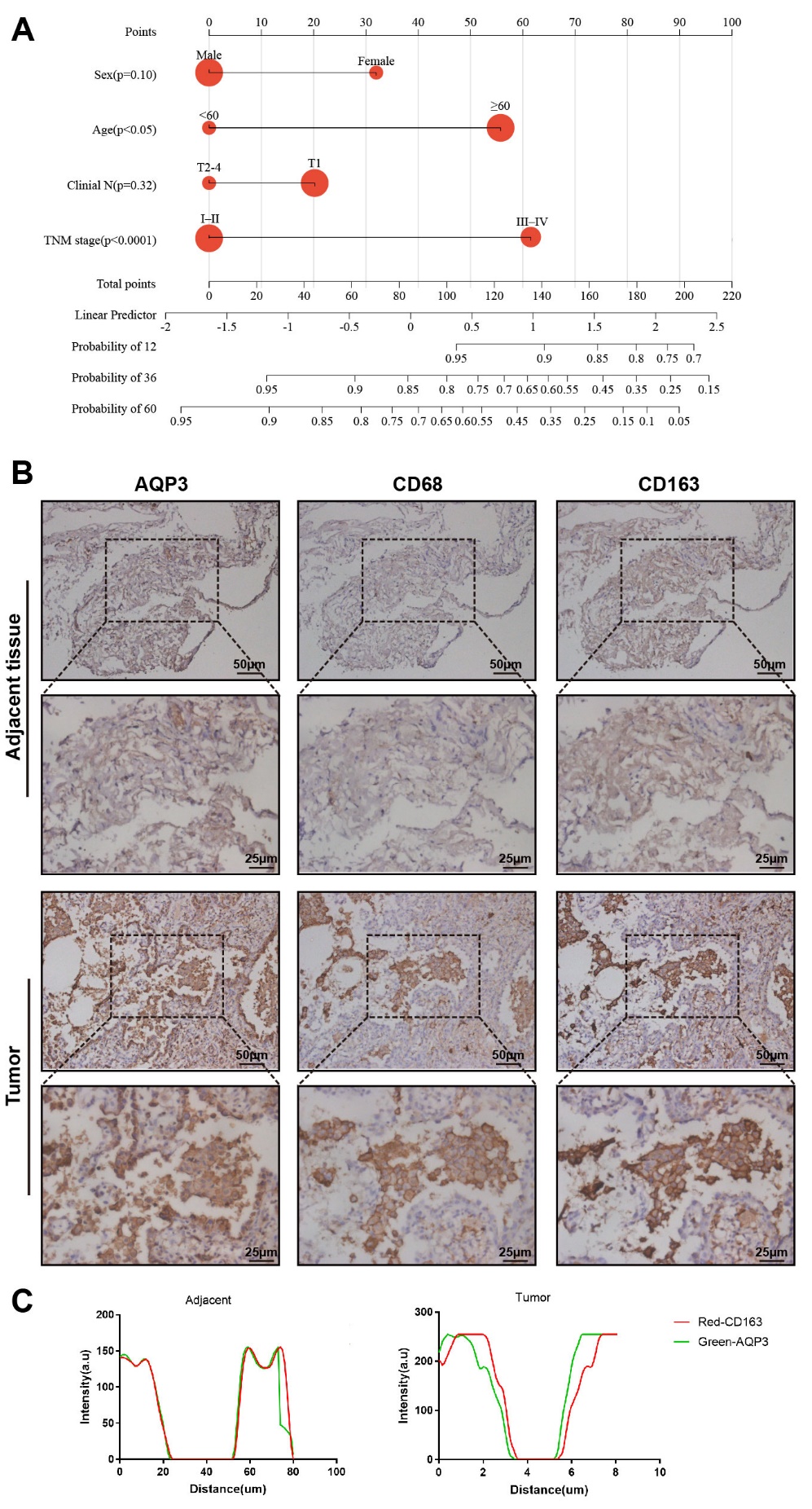


**Fig. S2 Prognosis prediction of AQP3 in LUAD and the expression correlation between AQP3 and macrophage markers.** (A) Nomogram was used to visualize the results of multi-cox regression analysis. (B) IHC staining was performed to show the expression of AQP3 between LUAD and adjacent tissues. (C) The fluorescence quantification analysis of CD163 and AQP3 expressions.


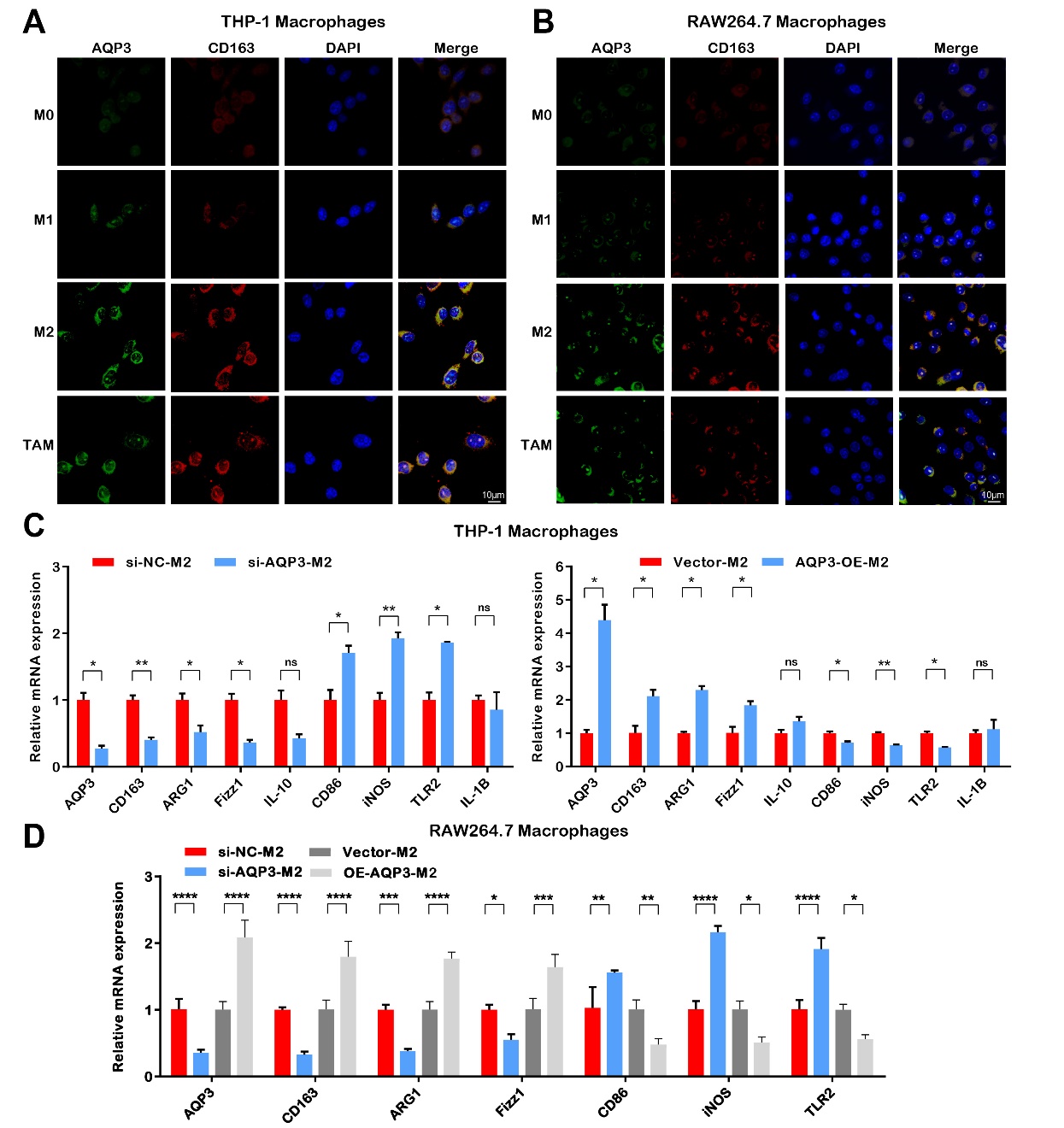


**Fig. S3 Relationship between AQP3 expression and M2 polarization**. (A, B) Immunofluorescent analysis of AQP3 and CD163 expressions in different types of macrophages. (C, D) RT-qPCR was performed to detect the AQP3 and CD163 expressions in THP-1 or RAW264.7 derived macrophages after AQP3 overexpression or knockdown.


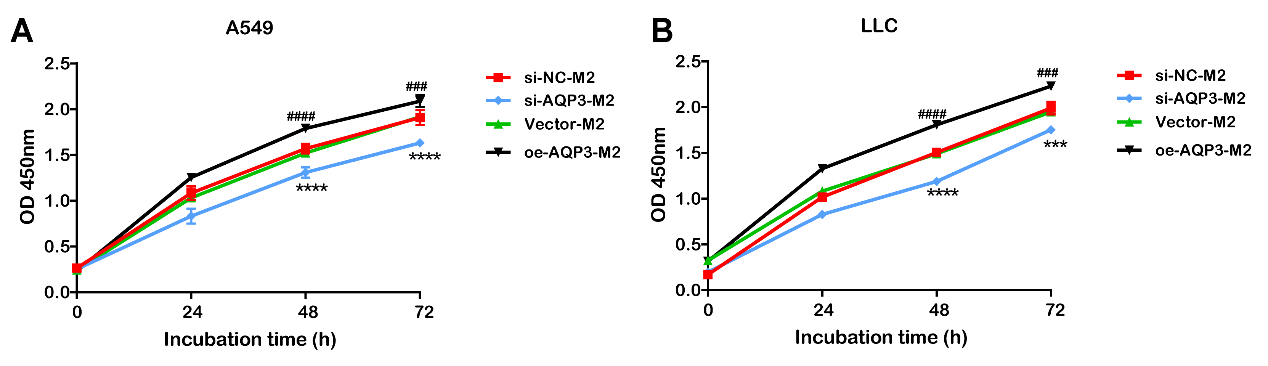


**Fig. S4** **CCK-8 assay showed the proliferation of A549 and LLC cells co-cultured with human and mouse-derived M2 macrophages with AQP3 knockdown or overexpression.**


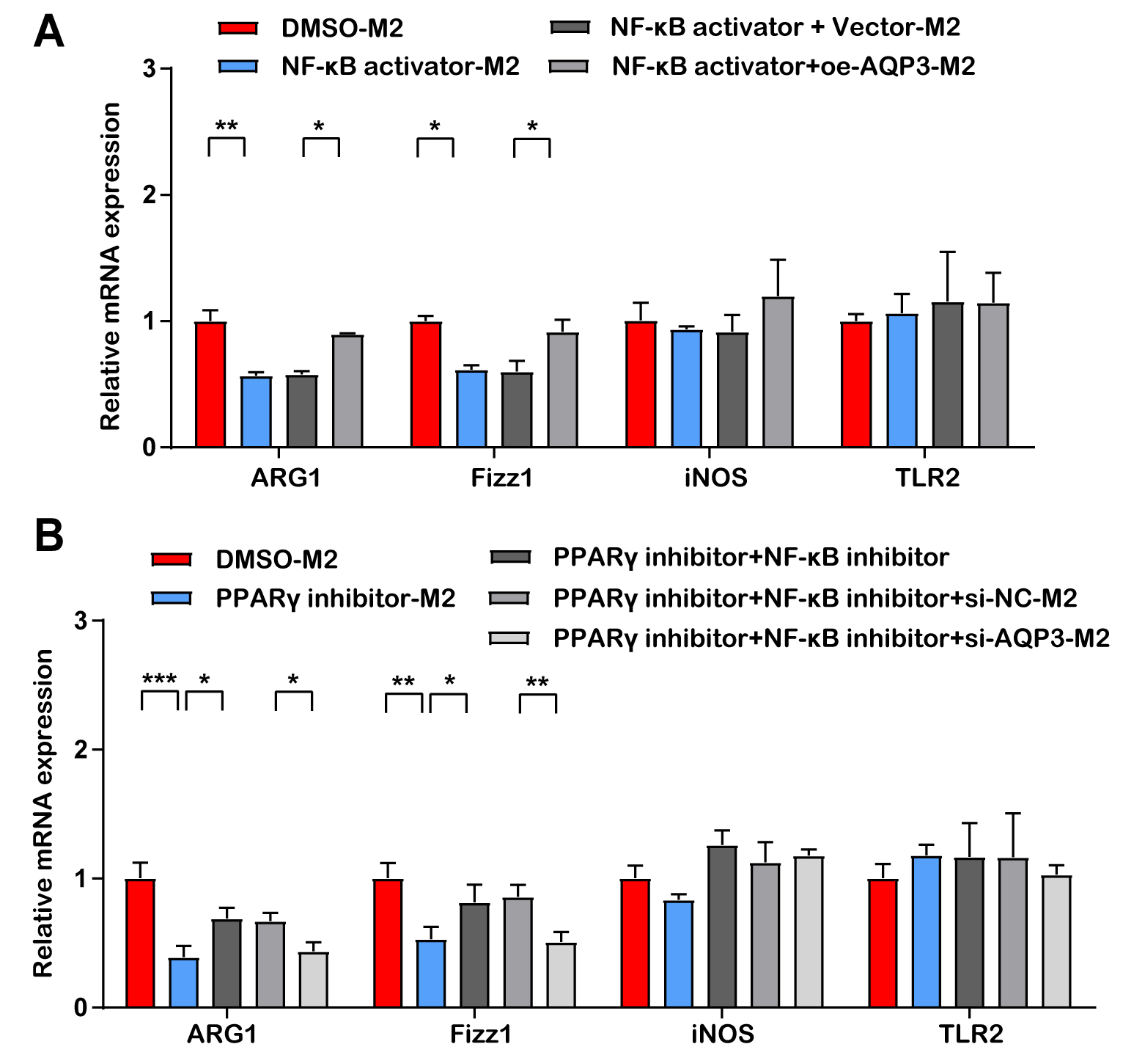


**Fig. S5** **The mRNA expressions of M2 macrophage markers altered by different intervention.** (A) The mRNA expressions of ARG1, Fizz1, iNOS and TLR2 in RAW264.7 derived macrophages after intervention of NF-κB activator or AQP3 overexpression. (B) The mRNA expressions of ARG1, Fizz1, iNOS and TLR2 in RAW264.7 derived macrophages after intervention of PPAR-γ inhibitor, NF-κB inhibitor or AQP3 knockdown.


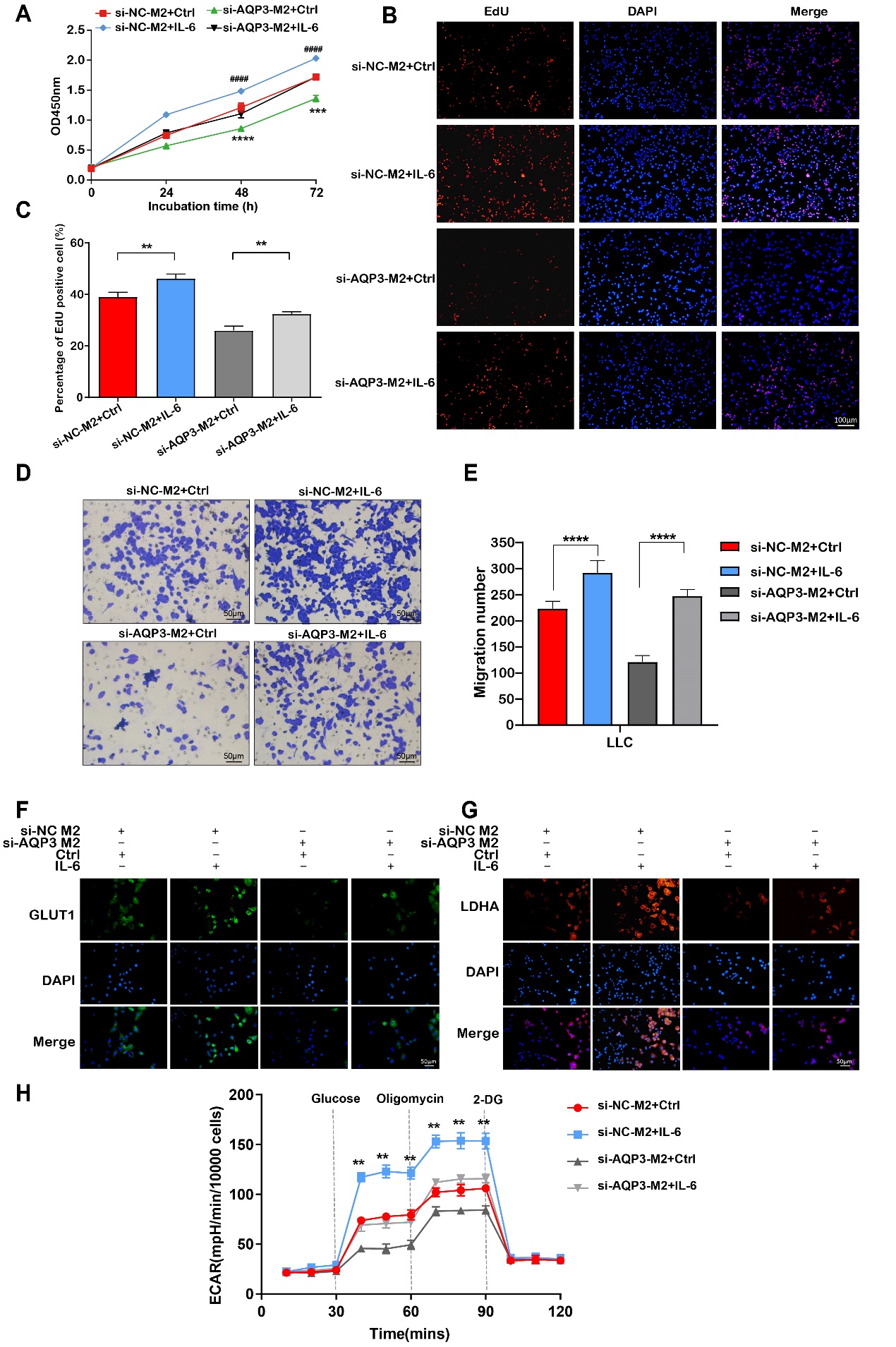


**Fig. S6 IL-6 mediated by AQP3 regulated proliferation, migration and glycometabolism of LUAD**. (A) CCK-8 assay showing the proliferation of LLC cells co-cultured with M2 macrophages after AQP3 knockdown and IL-6 stimulation. (B, C) Fluorescence image and bar graphs of EdU assay showing the proliferation of LLC cells co-cultured with M2 macrophages after AQP3 knockdown and IL-6 stimulation. (D, E) Transwell assay displaying the migration of LLC cells co-cultured with M2 macrophages after AQP3 knockdown and IL-6 stimulation. (F, G) Immunofluorescence staining of GLUT1 and LDHA showing the glucose metabolism of LLC cells co-cultured with M2 macrophages after AQP3 knockdown and IL-6 stimulation. (H) Aerobic glycolytic ability was measured using extracellular acidification rate (ECAR) assay in the co-cultured M2 macrophages.


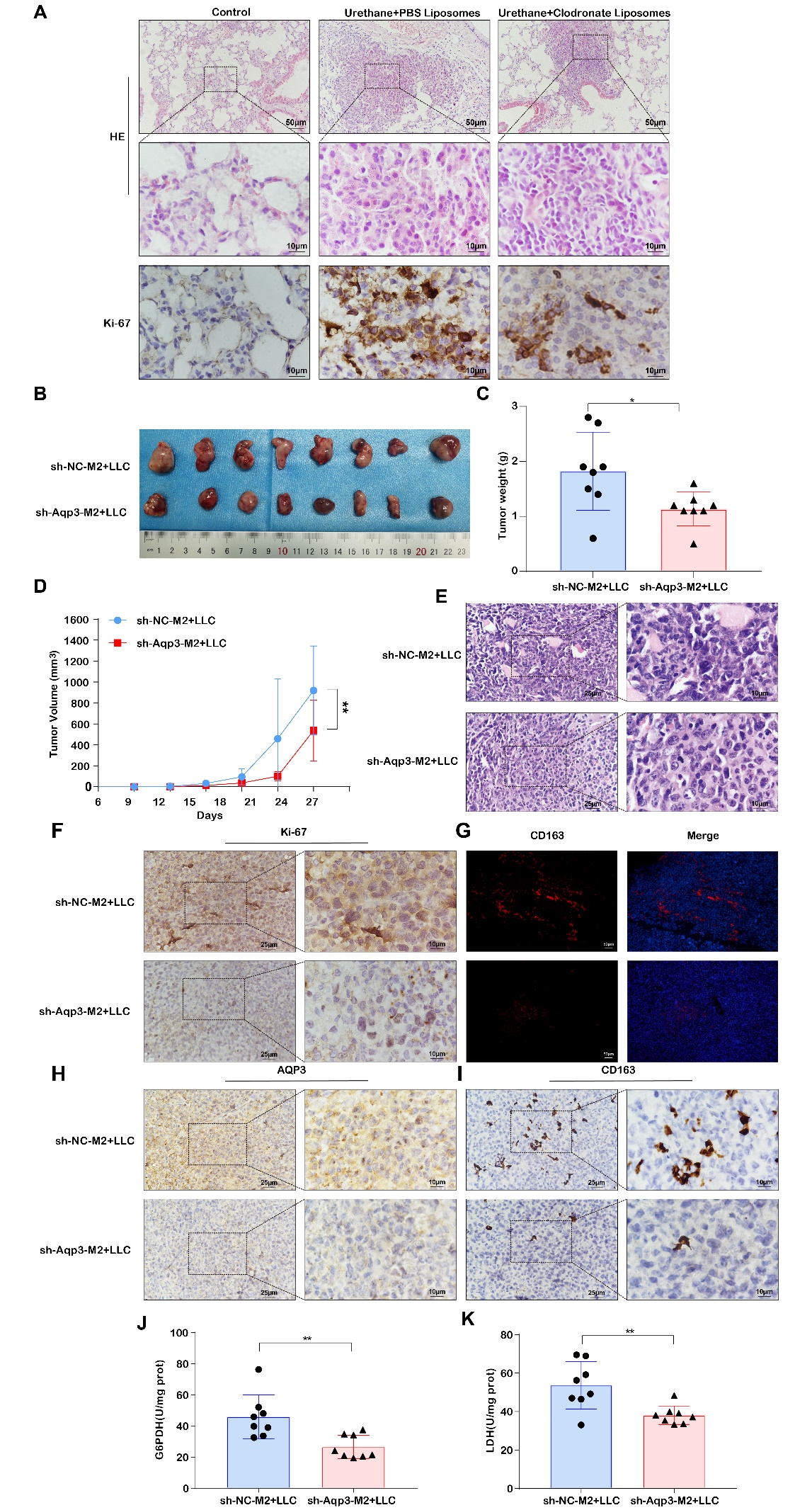


**Fig. S7 Clodronate liposomes and Aqp3 knockdown could suppress tumor progression *in vivo***. (A) HE staining and Ki-67 staining of lung nodule lesions in C57BL/6J mice under urethane and clodronate liposomes. (B) Representative images of co-inoculated subcutaneous tumors dissected from C57BL/6J mice after Aqp3 knockdown in macrophages. (C, D) The weight and growth curve statistics of subcutaneous tumors in the respective group. (E, F) HE staining and Ki-67 staining of subcutaneous tumors in different groups. (G-I) Immunofluorescence staining and IHC staining of CD163 and Aqp3 in subcutaneous tumors with different intervention. (J, K) The glucose metabolism of subcutaneous tumors was detected by G6PDH and LDH activity assays.


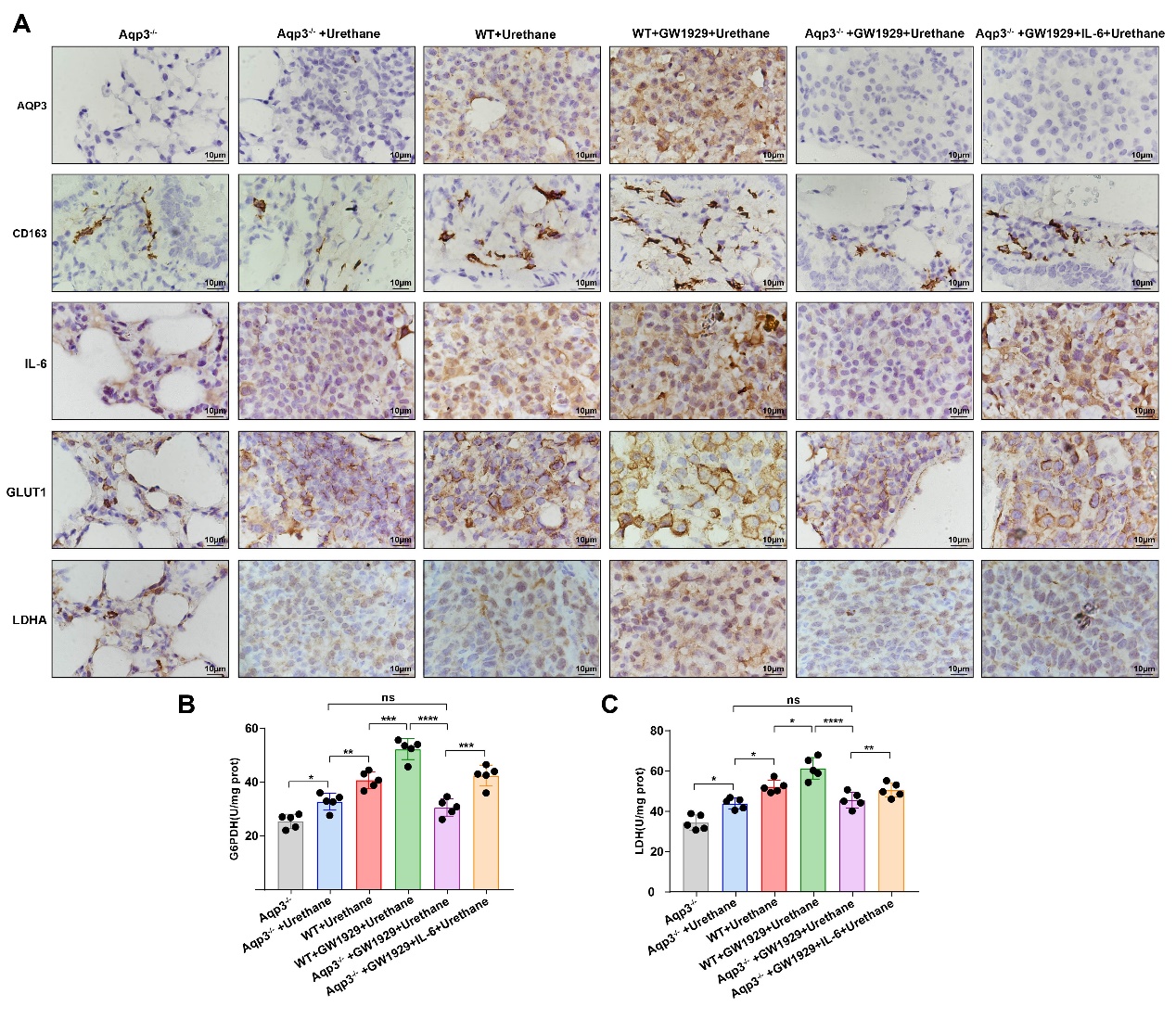


**Fig. S8 The effect of Aqp3 knockout mice in the urethane model.** (A) IHC staining showing the expression of AQP3, CD163, IL-6, GLUT1 and LDHA in Aqp3 knockout mice with different intervention. (B, C) The glucose metabolism of lung nodule lesions in the respective group was detected by G6PDH and LDH activity assays.
