# Supplementary material for: PPAR-γ/NF-kB/AQP3 axis in M2 macrophage orchestrates lung adenocarcinoma progression by upregulating IL-6
Source: Cell Death Dis. 2024 Jul 26;15(7):532. doi: 10.1038/s41419-024-06919-9 (PMC11282095; doi:10.1038/s41419-024-06919-9)

Original western blots

Fig 1B

GAPDH (patients 1-3)





AQP3 (patients 1-3)





GAPDH (patients 4-6 )





AQP3 (patients 4-6 )





GAPDH (patients 7-9 )


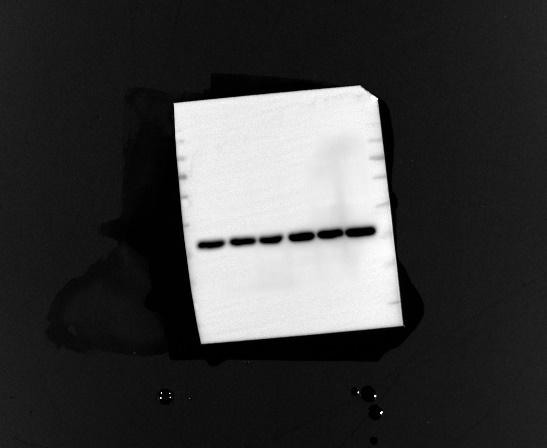


AQP3 (patients 7-9 )


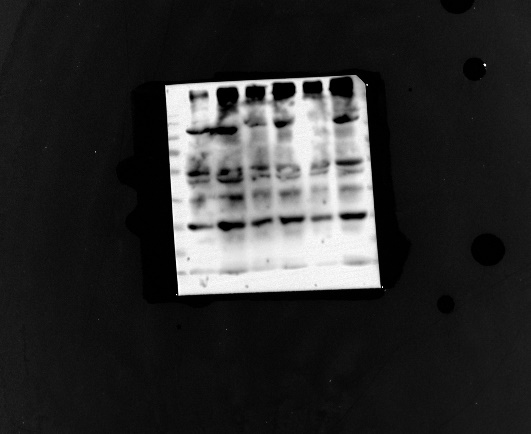


GAPDH (patients 10-12 )

­­
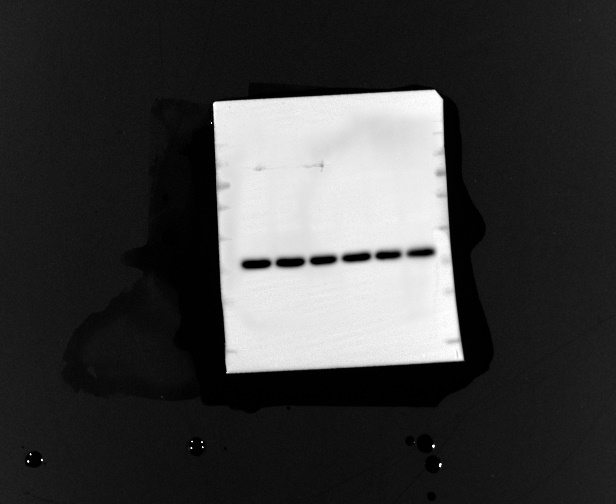


AQP3 (patients 10-12 )


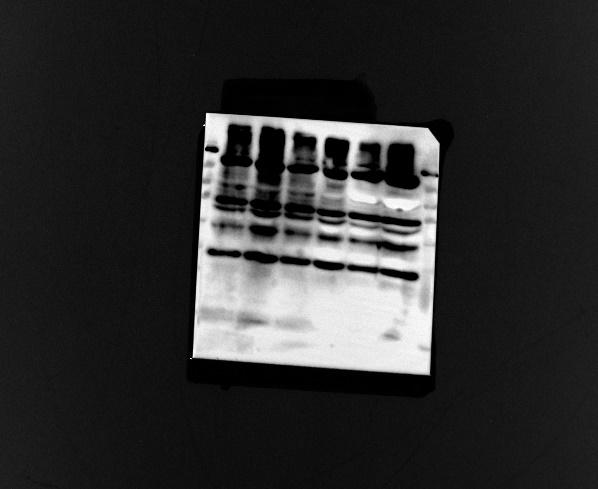


Fig 3C

GAPDH-cells





AQP3-cells





CD163-cells





Fig 3D

GAPDH





AQP3





CD163





Fig 6F

GAPDH





PPAR-γ





NF-Κb





p-NF-κB





IκB-α





p-IκB-α





Fig 6G

GAPDH





AQP3





CD163





PPAR-γ





NF-Κb





p-NF-κB





IκB-α





p-IκB-α





Fig 6H

GAPDH





AQP3


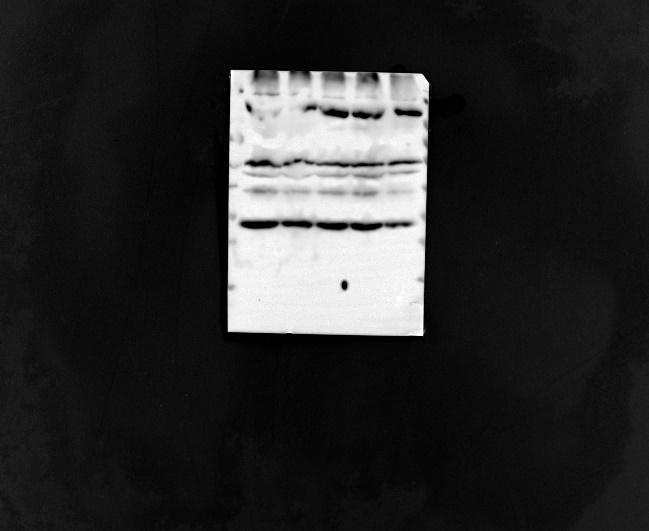


CD163





PPAR-γ





NF-κB





p-NF-κB





IκB-α





p-IκB-α





Fig 7A Cytokine antibody microarray image





Fig 7F

β-Actin


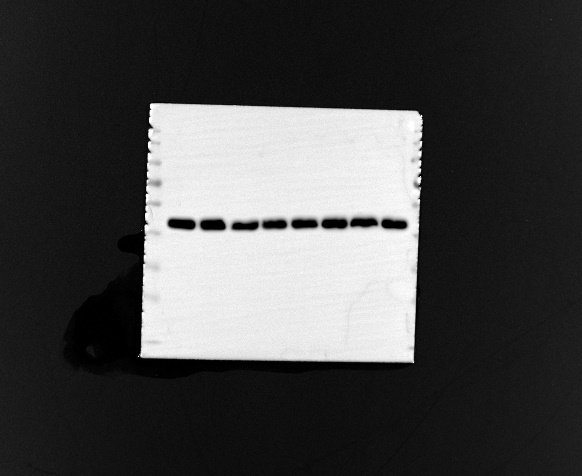


IL-6R


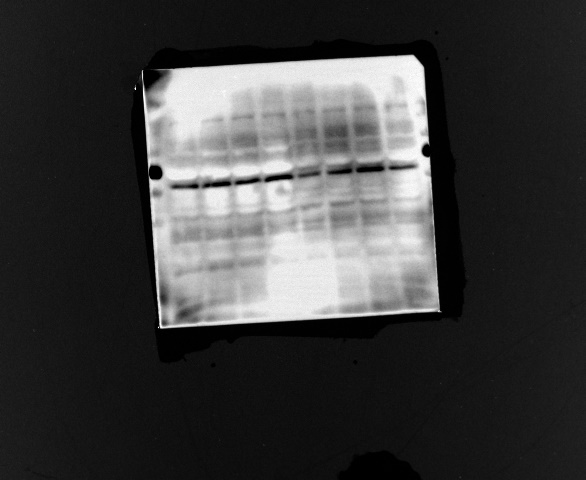


IL-6


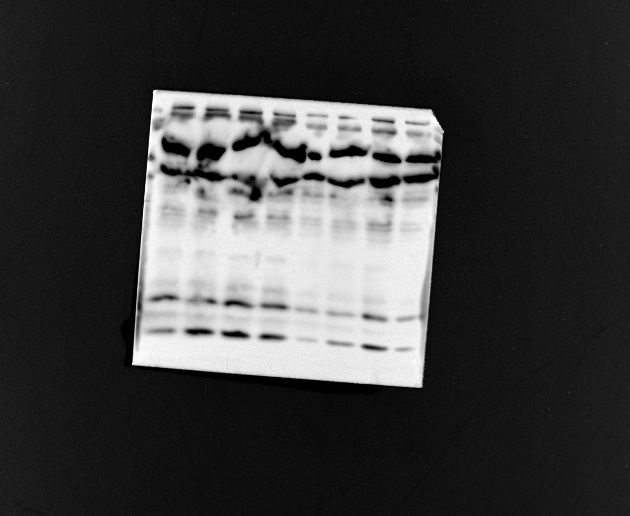


Fig 7G

β-Actin


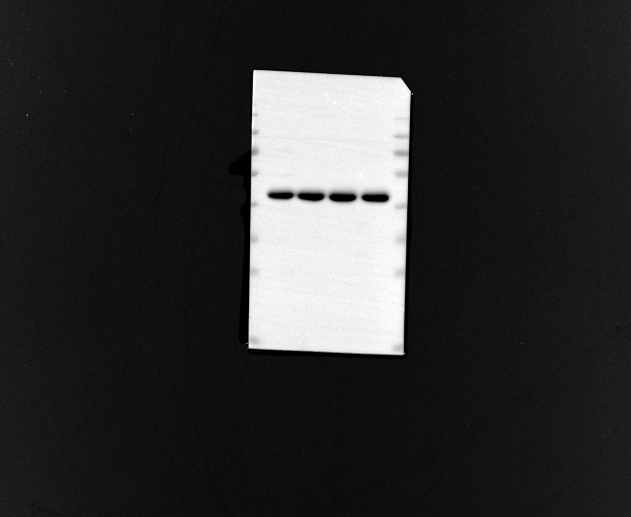


IL-6


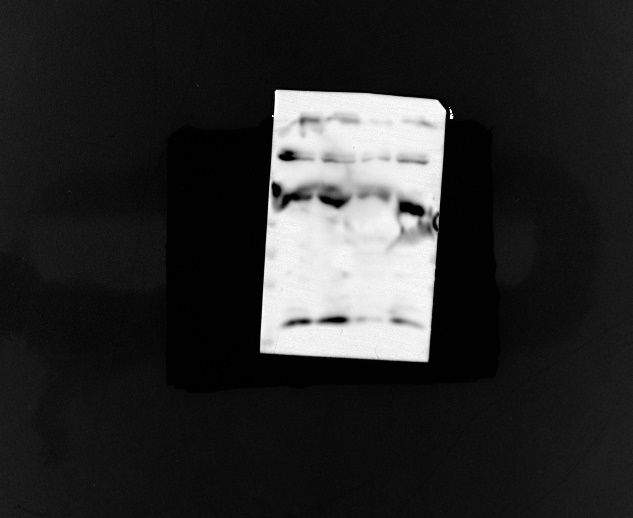


IL-6R


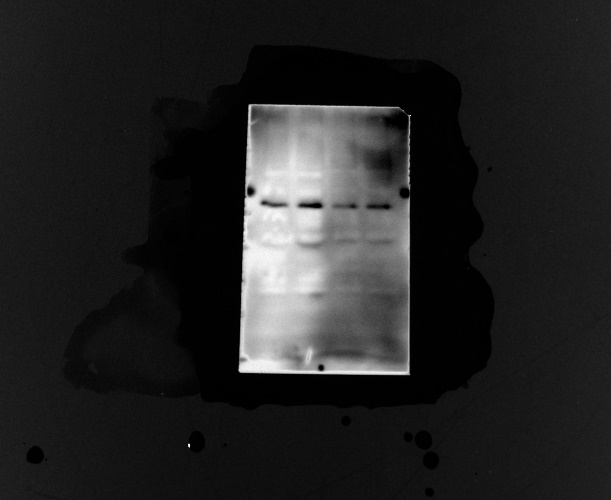


STAT3


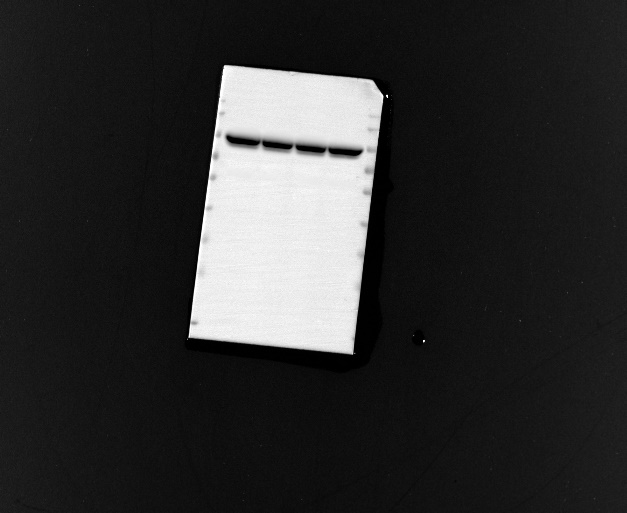


P-STAT3


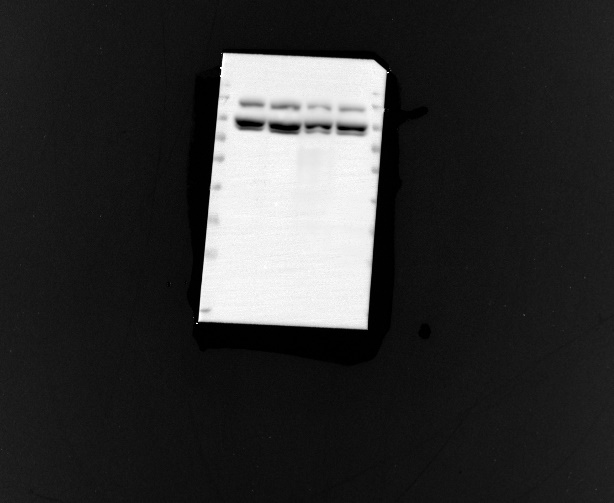

Supplement: Supplementary file 2 — Western blot original image [file 41419_2024_6919_MOESM2_ESM.docx]
